# Reviving grain quality in wheat through non‐destructive phenotyping techniques like hyperspectral imaging

**DOI:** 10.1002/fes3.498

**Published:** 2023-09-03

**Authors:** Luqman B. Safdar, Kateryna Dugina, Ali Saeidan, Guilherme V. Yoshicawa, Nicola Caporaso, Brighton Gapare, M. Jawad Umer, Rahul A. Bhosale, Iain R. Searle, M. John Foulkes, Scott A. Boden, Ian D. Fisk

**Affiliations:** ^1^ International Flavour Research Centre, Division of Food, Nutrition and Dietetics University of Nottingham Loughborough UK; ^2^ International Flavour Research Centre (Adelaide), School of Agriculture, Food and Wine and Waite Research Institute University of Adelaide Glen Osmond South Australia Australia; ^3^ Division of Plant and Crop Sciences, School of Biosciences University of Nottingham Loughborough UK; ^4^ Plant Research Centre, School of Agriculture, Food and Wine University of Adelaide Glen Osmond South Australia Australia; ^5^ Bühler UK Limited London UK; ^6^ Cotton Research Institute Chinese Academy of Agricultural Sciences Anyang China; ^7^ School of Biological Sciences University of Adelaide Adelaide South Australia Australia

**Keywords:** grain quality, hyperspectral imaging, plant breeding, wheat

## Abstract

A long‐term goal of breeders and researchers is to develop crop varieties that can resist environmental stressors and produce high yields. However, prioritising yield often compromises improvement of other key traits, including grain quality, which is tedious and time‐consuming to measure because of the frequent involvement of destructive phenotyping methods. Recently, non‐destructive methods such as hyperspectral imaging (HSI) have gained attention in the food industry for studying wheat grain quality. HSI can quantify variations in individual grains, helping to differentiate high‐quality grains from those of low quality. In this review, we discuss the reduction of wheat genetic diversity underlying grain quality traits due to modern breeding, key traits for grain quality, traditional methods for studying grain quality and the application of HSI to study grain quality traits in wheat and its scope in breeding. Our critical review of literature on wheat domestication, grain quality traits and innovative technology introduces approaches that could help improve grain quality in wheat.

## 1 INTRODUCTION

**FIGURE 1:**
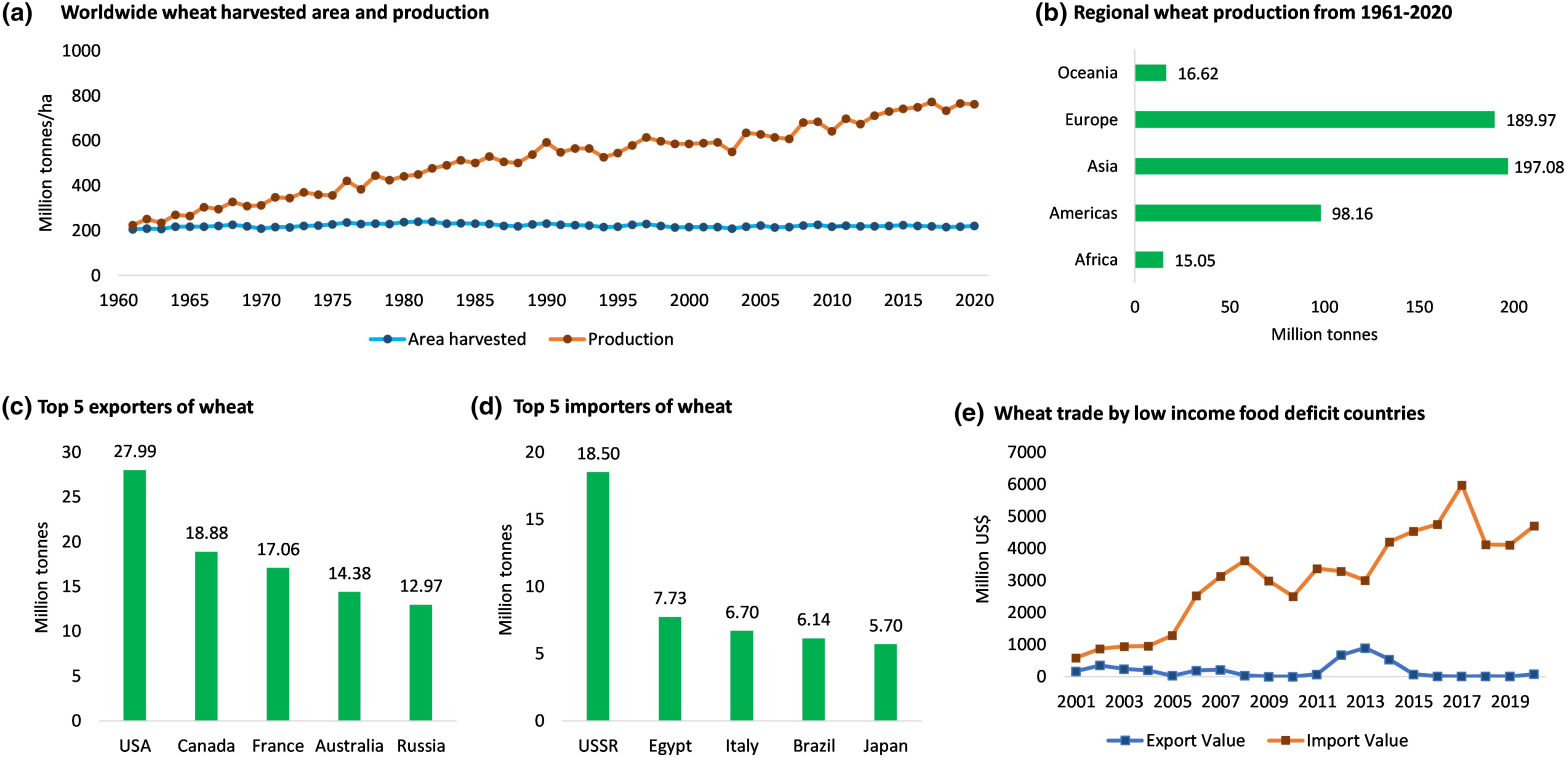
Wheat worldwide statistics from 1961 to 2020. (a) Wheat yield per unit area has significantly increased since 1960s given that the harvested area has not changed. (b) Asia and Europe produce more than 50% of the global wheat. (c) Top five wheat producing countries are all developed countries. (d) Top wheat importers are mostly developing countries which further puts a negative pressure on their economies. (e) In the last two decades, wheat imports by low‐income food deficit countries have massively increased whereas their exports have not changed. Figure is generated with data from FAOSTAT (FAO, 2022).

The world is facing a serious problem with the loss of 12 million hectares of arable land annually, primarily due to unsustainable agricultural practices. This issue is affecting almost 1 billion people in approximately 100 countries and threatening a large‐scale food crisis (GEF, 2022). Maintaining food security is a daunting challenge in the face of such a crisis. Wheat is an essential source of calories and protein for approximately 20% of the global population, with demand for wheat set to increase 60% by 2050. In low‐income food‐deficit countries, the gap between wheat export and import has increased significantly in the last two decades, with import values skyrocketing in recent years (Figure 1). This has led many developing countries to subsidise wheat products to stabilise prices, putting further pressure on availability and costs (Enghiad et al., 2017).

The demand for wheat is also increasing due to its high grain protein content and adaptability to grow in diverse environments (Reynolds et al., 2022). Although global wheat yield has increased in recent decades, the overall cultivated area has not (Figure 1). Traditionally, high wheat yield has been achieved through increased nitrogen fertilisation, but this has significant environmental costs, such as releasing harmful greenhouse gases into the atmosphere and damaging waterways and soil (Foulkes et al., 2009). Therefore, it is crucial to increase wheat yield per unit area of land without a commensurate increase in the use of nitrogen fertiliser. While increasing yield is critical, maintaining grain quality also presents significant challenges, as conventional methods for studying grain quality are destructive and labour‐intensive. Nonetheless, emerging non‐destructive high‐throughput phenotyping techniques, such as hyperspectral imaging (HSI)—an approach that combines near infrared spectroscopy and a broad‐spectrum camera to detect spectral and spatial information of objects—provide new opportunities for improving grain quality.

Recent reviews have focused on the use of HSI to investigate quality characteristics in cereals, including wheat (Caporaso et al., 2018b), wheat grain protein estimation (Ma et al., 2022), quality assessment at different stages of supply chain (Karmakar et al., 2022), application of HSI in plant phenotyping (Sarić et al., 2022) and comparison of HSI with near infrared spectroscopy to investigate quality characteristics (Tahmasbian et al., 2021). In contrast, we will discuss in this review how the loss of genetic diversity in modern wheat breeding could have led to selection against grain quality and key traits that influence the nutritional value of wheat. Additionally, we will discuss techniques used commonly to study grain quality along with their limitations and introduce HSI as an emerging high‐speed non‐destructive technique that can improve the capability of plant breeding to improve grain quality while sustaining yield gains. In doing so, we aim to provide a unique resource that improves our understanding of how traditional grain quality phenotyping methods can be replaced by non‐destructive techniques that could help improve wheat grain quality.

### 1.1 Loss of genetic diversity during domestication and modern wheat breeding

The domestication of wheat started nearly 10,000 years ago with the diploid einkorn and tetraploid emmer, and today the most widely grown wheat includes *Triticum durum* (Maccaferri et al., 2019), while the most widely cultivated hexaploid wheat is *T. aestivum*, which was created through hybridisation of wild emmer with *Aegilops tauschii* (Matsuoka & Nasuda, 2004; McFadden & Sears, 1946). While domestication led to the selection of favourable traits for cultivation, a major loss in genetic diversity occurred during modern breeding. Breeding focused on selecting genes that influence traits of agricultural value such as free‐threshing grain, plant architecture, vernalisation, photoperiod‐dependent flowering and grain protein content, resulting in reduced genetic diversity of modern wheats. Recent research revealed that modern bread wheat varieties have lost an average of 21.8% nucleotide diversity over the past two centuries of breeding improvement, with the loss distributed randomly among the A, B and D sub‐genomes (Pont et al., 2019). The Green Revolution, which introduced *Reduced height* (*Rht*) genes that result in shorter plants with increased grain production may have contributed to this diversity loss (Smale, 1997), potentially limiting the genetic potential for improving other key traits, including grain quality. Therefore, it is essential to explore and utilise diverse germplasms to enhance crop quality and production. Global collaborations such as the Global Durum Wheat Panel (Mazzucotelli et al., 2020), recently sequenced collection of D subgenome progenitor *A. tauschii* ranging from Western Asia to China (Gaurav et al., 2022), and the global A. E. Watkins landrace collection (Miller et al., 2001) provide opportunities for improving wheat grain quality by incorporating diverse germplasms.

## 2 KEY GRAIN QUALITY INDICATORS IN WHEAT

Wheat is the oldest and most important cereal crop, with bread wheat used for flour and durum wheat for pasta. Nearly 20% of the total caloric and protein intake worldwide relies on wheat‐based products, making it a significant source of carbohydrates, proteins, fats, fibres, essential mineral nutrients and vitamins (FAO, 2022). Improving quality of wheat grains is, therefore, critical as they provide a significant proportion of the global caloric needs. In this section, the phenotypic traits that play key roles in determining the grain quality and their nutritional value will be discussed.

**FIGURE 2:**
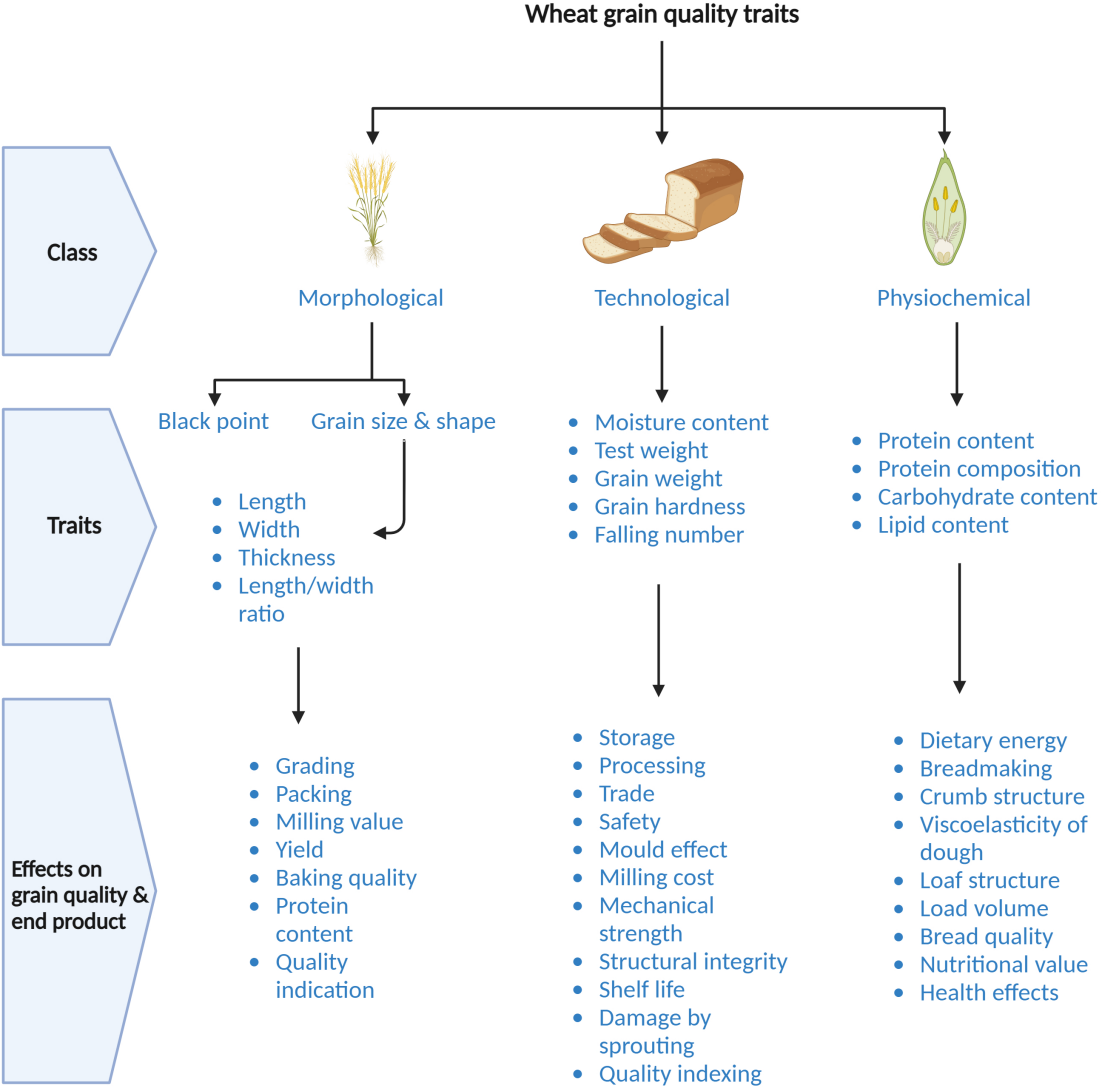
Wheat grain quality traits. The factors which determine wheat grain quality can be classified into morphological, technological and physiochemical characteristics. The figure demonstrates different parameters involved in each category and how they affect the grain in terms of quality, milling performance, yield stability and nutritional and health properties. For simplification, all the effects of each category have been presented together, for example, the effects of gain length, width, thickness and length/width ratio have been put collectively under the category of morphological characteristics. Their details are described in the text separately (Created with BioRender.com).

Grain quality is determined by a range of characteristics, which can be broadly classified into morphological, technological and physiochemical indicators (Figure 2). Technological and physiochemical indicators, such as grain protein content and Hagberg falling number, are particularly important because they play a significant role in determining the rheological properties, such as viscosity, elasticity and extensibility of flour and dough.

### 2.1 Morphological indicators of grain quality

A typical wheat grain is between 4 and 8 mm in length and weighs 35–55 mg, consisting of the pericarp, aleurone layer, endosperm, and germ or embryo. Flour extraction and quality are determined by the ratio of these components. The milling process involves removing the aleurone and pericarp to form bran, then removing the germ to produce white flour from the pure endosperm. Therefore, it is crucial to consider the morphological indicators of wheat quality such as grain size and shape to ensure the milling process is efficient.

Larger grain size and spherical shape are desirable features that have been selected for in modern cultivars (Gegas et al., 2010). Grain size is also associated with chemical characteristics of flour, such as protein content and hydrolytic enzyme activity, which affect baking quality and end‐use suitability (Evers, 2000). Larger grains have a smaller husk fraction and therefore high percent protein.

Black point is a dark discolouration at the germ end of the grain and is considered a negative indicator of quality. The causes are not fully established, but it has been associated with abiotic stresses such as high humidity or extreme temperatures during grain fill (Clarke et al., 2004; Kumar et al., 2002) or fungal infections (Conner & Kuzyk, 1988; Jacobs & Rabie, 1987). However, a study of 12 grain samples reported that black point had no significant effect on the baking or bread‐making quality (Rees et al., 1984).

### 2.2 Technological indicators of grain quality

Grain quality indicators such as moisture content, test weight, grain weight and grain hardness are vital for the food industry, as they determine the storage potential, yield, durability and crumb structure of bread. These traits are genetically regulated and heritable (Barnard et al., 2002; Taneva et al., 2019) and are the focus of breeding programs for their improvement.

Moisture content is an essential quality indicator in grain trading, storage, processing and when comparing the grade of different samples. Either a maximum or a range of moisture content is stated in trading contracts, and cost penalties may be incurred if it falls outside specified levels. The International Organization of Standardization (ISO 7970‐2021) sets a limit of 14.5% for wheat grain moisture content. However, different moisture contents may be required for specific destinations, depending on climate, transportation duration and storage conditions. Moisture content above 18% can significantly reduce the number of weeks for mould‐free grain storage (Gedye et al., 1981). Besides storage, moisture content affects the mechanical properties of grains and production costs (Ahmed et al., 2015). Higher moisture content strengthens the gluten network and enhances its sorption capacity (Warechowska et al., 2016).

Test weight is the weight per specific volume of wheat and is often used as a quality indicator for soft winter wheat, as it can predict potential flour yield. However, various factors, including moisture content, grain size, damage, shrunken or broken grains, wetting and drying, and the milling process, can affect the test weight (Schuler et al., 1995). It is, therefore, not a reliable indicator of flour yield or milling quality. The test weight of wheat varies depending on the climate and region of growth.

Grain weight is a complex trait influenced by genetic and environmental factors. It is an important indicator of quality and an integral yield component. The genetics of grain weight are complex, as it is a polygenic trait comprising many subcomponents, including grain length, width, height, filling rate and carpel size (Brinton & Uauy, 2019). The connection between carpel size and final grain weight is based on the fact that potential grain weight is related to the size of the ovary (Reale et al., 2017). Grain weight shows variation across different genotypes, within a single genotype or even within a single spike. It is a more accurate guide of flour yield than test weight as it shows the efficiency with which a grain has been filled (Wang & Fu, 2020).

Grain hardness is an essential quality indicator that relates to the endosperm structure and the way it breaks during milling. Endosperms of hard wheat offer considerable resistance to the crushing action of mill rolls, retain a discrete granular shape of particles that have uniform sizes, and provide a free‐flowing flour that is easily sieved. By contrast, soft endosperm breaks up easily into irregular particles that vary widely in size (Turnbull & Rahman, 2002). Grinding hard wheat requires higher energy costs due to an increase in grinding energy consumption (Dziki & Przypek‐Ochab, 2009).

The Hagberg Falling number (HFN) is a significant indicator of grain quality in wheat and other cereals, widely used in worldwide grain trade. It assesses flour quality and estimates damage caused by excessive α‐amylase activity from preharvest sprouting (Hagberg, 1960). The HFN is typically determined by creating a slurry of flour and water with a known ratio, and then measuring the density of the mixture indirectly. This is done by dropping a metal object of known weight into the mixture and timing how long it takes to reach the bottom of the container. Shorter times indicate higher degrees of starch hydrolysis, which can have a negative impact on breadmaking quality (Newberry et al., 2018). High α‐amylase is associated with sticky dough and poor crumb structure (Kim et al., 2006). The enzyme hydrolyses long‐chained starch molecules into simpler glucose and maltose sugars, which occurs due to excess rainfall signalling the embryo to germinate (Kandra, 2003). External factors, particularly rainfall, affect α‐amylase activity and ultimately HFN. Grains with low HFN have lower test weight and are considered damaged grains (Halverson & Zeleny, 1988). Wheat samples with HFN >350 s are preferred as quality samples, while those with HFN <250–275 s are considered damaged and often discounted (Hareland, 2003). Care should be taken while selecting samples from batches for estimating HFN because batches are typically bimodal. For instance, in a study of 425 wheat samples, 53 samples had HFN <150 s while 372 had >150 s (Caporaso et al., 2017). Therefore, emerging phenotyping techniques that evaluate heterogeneity across single grains, such as hyperspectral imaging, become of great importance.

In conclusion, technological indicators of grain quality play an essential role in the food industry. Grain moisture, weight, hardness and falling number are key parameters for quality control and indicate storage potential, yield potential, durability and crumb structure of bread. However, these traits are influenced by genes and the environment, and storage conditions, temperature and rainfall can affect the quality of the grain. Improving these technological traits through breeding and genetic modification is essential to maintain and improve quality and ensure a consistent supply of high‐quality grain for the food industry.

### 2.3 Physiochemical indicators of grain quality

**FIGURE 3:**
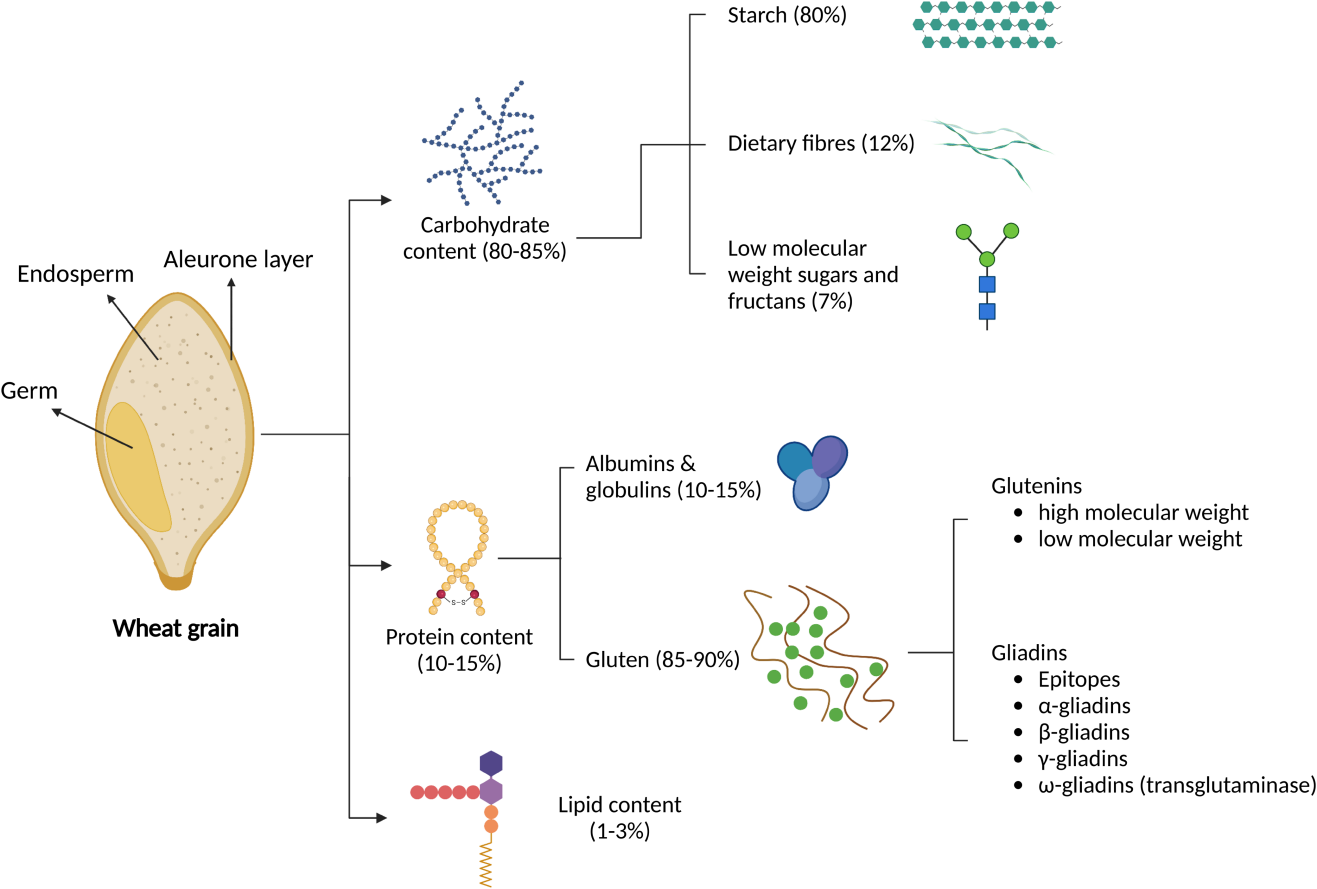
An illustration of wheat grain and its physiochemical properties. A mature grain contains ~85% carbohydrates and ~10–15% proteins. The majority proportion of carbohydrates is starch and that of proteins is gluten. High protein wheat often comes with a trade‐off of low starch. Lipid content is present in a very small quantity (1–3%) in the germ part of the grain (Created with BioRender.com).

Wheat grains are made up of 85% carbohydrates and 10–15% proteins, with 80% of the carbohydrates being starch. The other 15% is composed of low molecular weight sugars and fructans, as well as dietary fibres (Shewry & Hey, 2015) (Figure 3). These components are important in determining wheat grain quality.

Starch is made up of two glucose polymers, and it is the main source of dietary carbohydrates. It plays an important role in breadmaking, as wheat, barley and rye starches have similar properties that produce satisfactory bread (Hoseney et al., 1969). The physicochemical properties of starch, such as crystallinity and granule size distribution, can affect the quality of bread (Cauvain, 2012). Some of the starch escapes digestion in the small intestine, known as resistant starch, which is associated with a reduced risk of colorectal cancer (Humphreys et al., 2014) and insulin sensitivity (Lobley et al., 2013).

Dietary fibres such as arabinoxylan, β‐glucan, fructans, lignin and resistant starch (Stone & Morell, 2009) are cell wall polysaccharides present in the pericarp of wheat. They help to prevent a variety of diseases, including blood pressure, hypertension, type‐2 diabetes, stroke, constipation, colorectal cancer and colon cancer (Shewry & Hey, 2015). Wholegrains are a good source of dietary fibre, vitamins, minerals and phytochemicals, which can contribute to protective effects as compared to refined grains (Slavin, 2003). The composition of dietary fibres differs between wholegrain and white flour, with the latter containing mostly arabinoxylan and β‐glucan (Andersson et al., 2013).

Lipids are present in wheat grain, usually in minor quantities (2–4%), and are concentrated in the germ. They can be broadly separated into two classes, nonpolar and polar lipids, both of which are present in flour in roughly equal quantities. Nonpolar lipids are considered to have a negative effect on loaf volume, while polar lipids are thought to be beneficial to bread quality (Pyler & Gorton, 2008). As lipids are very surface‐active compounds, they can be involved with gas bubble stabilisation mechanisms in dough, but it is unclear if their surface‐active nature is competitive enough in dough making (Cauvain, 2012).

Grain protein content (GPC) is important for the quality of bread and pasta, and typically makes up 10–15% of the grain's dry weight (Shewry & Hey, 2015). Protein quantity as well as quality are crucial for breadmaking, as both polymeric proteins (glutenins) and monomeric proteins (gliadins) contribute to dough's viscoelastic properties. Studies have shown that higher protein content improves the quality of bread and pasta, as it makes the dough more cohesive and stronger, able to hold more carbon dioxide (Cauvain, 2012). While all components of grain contribute to flour and product value, protein quantity and quality remain major factors for high‐quality bread. However, efforts to increase GPC have led to lower grain yields, which is undesirable for breeding programs. Some increase in GPC can be achieved through increasing nitrogen fertiliser application, but this strategy is not only expensive but can contaminate the soil (Giles, 2005) and raise health concerns (Ward, 2009). Despite progress in understanding the genetic basis of GPC regulation (Distelfeld et al., 2004; Uauy, Brevis, & Dubcovsky, 2006; Uauy, Distelfeld, et al., 2006), not many commercial cultivars with desirable GPC and amino acid profile are introduced. Deviation from the negative relationship between GPC and yield, also known as grain protein deviation (GPD), has been proposed as a potential criteria to select yield and GPC simultaneously (Bogard et al., 2010), with some promise shown in hybrid wheat (Thorwarth et al., 2018). However, research into GPD is still limited and therefore, producing cultivars with GPC and yield balance remains a challenge for breeders.

In conclusion, the physiochemical indicators of grain quality, including starch, dietary fibres, lipids and protein content, are crucial in determining the quality of grain and its products. Although there have been attempts to increase protein content, the quality of protein is vital for improving bread quality. In the following section, we will delve into the major proteins found in wheat and their roles in the structural and nutritional properties of bread.

## 3 MAJOR PROTEINS IN WHEAT GRAIN

Wheat proteins can be classified into albumin, globulin and gluten based on their solubility in different aqueous solutions (Shewry, D'Ovidio, et al., 2009). Gluten proteins constitute 85–90% of the total proteins while albumin and globulin make up the remaining 10–15% (Figure 3).

### 3.1 Gluten proteins in wheat and their role in breadmaking

Gluten proteins, comprising gliadin and glutenin, play key roles in grain quality (Shewry, 2019). Gliadin generally occurs as a heterogeneous mixture of single‐chain polypeptide subunits (Wieser, 2007), while glutenin occurs as multi‐chained proteins (Wieser et al., 2006). The structure of gliadins contains an N‐terminal domain with repetitive amino acid sequences rich in proline, glutamine and phenylalanine, and a C‐terminal domain (Grosch & Wieser, 1999). Both gliadins and glutenins are enriched for proline and glutamine residues and are generally referred to as prolamins (Wieser et al., 2022). Cysteine residues are also important structural components of both these proteins, being involved in intramolecular or intermolecular disulphide bonds (Veraverbeke & Delcour, 2002). Glutenins are too large to be separated by gel electrophoresis, but the disulphide linkages can be reduced by treating glutenins with β‐mercaptoethanol or dithiothreitol, which yields less‐complex low‐ and high‐molecular glutenin subunits (LMW‐GS and HMW‐GS) soluble in aqueous ethanol (Veraverbeke & Delcour, 2002).

Gluten proteins determine the breadmaking quality of wheat flour by providing cohesivity, viscosity and elasticity to the dough when hydrated. Gliadin contributes to viscosity and extensibility, while glutenin provides strength and elasticity to the dough system (Biesiekierski, 2017; Wieser, 2007). The quality of gluten proteins is influenced by the composition, structure and interaction of their subclasses (Veraverbeke & Delcour, 2002). An imbalance between viscosity and elasticity negatively affects dough rheological properties, leading to low bread loaf volume (Shewry et al., 2002). Thus, achieving a balance between gliadins and glutenins is crucial for optimal gluten rheological properties and breadmaking quality. The addition of oxidants, reducing agents or proteases to the flour can modify gluten rheological properties. It is important to note that other flour components, such as arabinoxylans, flour lipids and nongluten proteins, can also impact dough rheological properties (Chung, 1986).

Increasing GPC can improve breadmaking quality as it increases the fraction of gluten proteins more compared to non‐gluten proteins (Hoseney, 1994). Studies have been conducted to explore genes regulating the synthesis of gliadins (Gao et al., 2007) and glutenins (Payne & Lawrence, 1983) and the interaction of dough quality traits and genetic variation for gluten proteins in wheat. More recently, quantitative trait loci (QTLs) associated with dough quality traits have been identified (Pshenichnikova et al., 2008; Ruan et al., 2020); however, more research is needed to understand the molecular mechanisms underlying the formation of gluten proteins and their interactions in dough.

### 3.2 Non‐gluten proteins

Non‐gluten proteins in wheat, mainly albumin and globulin, are present in smaller amounts and are generally monomeric. These proteins have various metabolic functions, including plant defence and storage (Carbonero & García‐Olmedo, 1999). Non‐gluten proteins contribute to nearly 50% of the lysine content in wheat (Fra‐Mon et al., 1984). Lysine is the first essential amino acid in grain (Shewry & Hey, 2015) and boosting its content has been a breeding target for over 50 years (Shewry, 2007). Although gluten proteins are the main determinant of bread quality, non‐gluten enzymatic proteins such as proteases (Bleukx et al., 1998), xylanases (Cleemput et al., 1997), protease inhibitors (Goesaert et al., 2006) and xylanase inhibitors (Debyser & Delcour, 2008) have been reported to impact breadmaking. However, the role of non‐gluten proteins in flour's breadmaking properties is not well understood.

### 3.3 Critical stages for protein deposition

Studies have been conducted to observe the patterns of protein deposition at various stages of grain development. Protein deposition in grains occurs largely in sub‐aleurone cells, with the majority happening between 14 and 35 days after anthesis (DAA) (Shewry, Underwood, et al., 2009). Structural proteins (albumins and globulins) accumulate up to 25 DAA, followed by storage proteins (gliadin and glutenin fractions) (Stone & Savin, 1999). Thus, early to middle stage of grain development, or grain filling, is the most critical time for protein deposition.

Once the key traits that can influence grain quality and breadmaking of flour have been identified, it is important to consider the available approaches to study these traits. Accurate phenotyping is critical to study and improve a trait of interest. Therefore, in the next section, we will discuss the approaches used commonly to study grain quality traits in wheat and investigate newly emerging techniques that could accelerate the process of quality improvement.

## 4 METHODS FOR STUDYING GRAIN QUALITY CHARACTERISTICS

Common approaches for studying grain quality traits involve destructive and non‐destructive techniques. Destructive techniques involve breaking down samples into non‐reusable ground matter or liquid solvents, whereas non‐destructive techniques allow for further analysis without destroying the samples. Techniques used to study grain chemical, structural and mechanical properties include microscopy, nitrogen estimation, chromatography with spectrometry and spectroscopy.

### 4.1 Microscopy techniques

Microscopic techniques are used to study the grain structure, which varies in features such as cell size, wall thickness, starch and protein distribution and structure, and lipid content. Several microscopy techniques are available, including transmission electron microscopy (TEM), scanning electron microscopy (SEM), confocal laser scanning microscopy (CLSM), atomic force microscopy (AFM) and X‐ray computed tomography (X‐ray CT). Examples of their recent use to investigate grain quality traits include starch granule visualisation by TEM (Hawkins et al., 2021), effect of α‐amylase activity on starch by SEM (Roy et al., 2013), interaction between dietary fibres and wheat gluten by CLSM (Li et al., 2017), alterations in protein‐starch interface due to grain hardness by AFM (Chichti et al., 2015) and wheat spike architectural traits using X‐ray CT (Zhou, Riche, et al., 2021). Each technique has its advantages and limitations. The use of these techniques has allowed for the observation of various aspects of grain structure, such as protein variations, degradation of starch, structural differences of gluten and alterations in protein‐starch interface. These observations can contribute to understanding the quality of the grain and the product derived from it.

### 4.2 Methods for estimating nitrogen content: Kjeldahl and dumas

In 1883, Johan Kjeldahl, a Danish chemist, developed the Kjeldahl method, which has since been used widely to estimate nitrogen and protein content in various species (reviewed elsewhere (Sáez‐Plaza et al., 2013)). The method involves estimating the nitrogen content, which is then multiplied by a nitrogen‐to‐protein conversion factor of 6.25 to predict the protein content (Mariotti et al., 2008). For wheat, a conversion factor of 5.7 is used as it depends on the average amino acid composition of the species analysed (O'Sullivan et al., 1999). It is important to note that predicted protein represents the overall protein content and not the structural or functional types (Sáez‐Plaza et al., 2013). The disadvantage of Kjeldahl method is it can only measure nitrogen bound to free amino acids, nucleic acids, proteins or ammonium, and not from other forms like nitrates or nitrites in the sample.

An alternative to the Kjeldahl method is the Dumas combustion method, which converts all forms of nitrogen in a sample to nitrogen oxides through combustion at 800–1000°C. The nitrogen oxides are then reduced to N_2_, and the N_2_ is measured by a thermal conductivity detector. The entire process takes approximately 5 min per sample and is safer as it circumvents the use of hazardous chemicals (Müller, 2017). Unlike the Kjeldahl method, the Dumas method measures nitrogen from all organic and inorganic sources (Simonne et al., 1997) and is, therefore, recommended for measuring nitrogen from plant materials containing high amounts of nitrogen associated with nitrates or nitrites (Watson & Galliher, 2001).

### 4.3 Chromatographic and spectrometric techniques

Chromatographic and spectrometric techniques are commonly used in cereal research for the separation and identification of different compounds in a mixture. Gel permeation chromatography (GelPC), high performance liquid chromatography (HPLC), high performance anion exchange chromatography (HPAEC), gas chromatography (GC) and mass spectrometry (MS) coupled with GC & LC are some examples of these techniques. They have been used for various purposes, including the examination of amylose structure and purity (Takeda et al., 1986), the determination of gluten protein types in flour (Wieser et al., 1998) and the quality assessment of durum varieties (Hailu et al., 2016). Nanoscale secondary ion mass spectrometry (NanoSIMS) is a technique used to map the elemental and isotopic composition of a sample cross section at nanoscale resolution with high sensitivity. NanoSIMS has been used to analyse tissue distribution of selenium and arsenic (Moore et al., 2010) and iron (Sheraz et al., 2021) in grains, revealing differential mineral concentrations between the aleurone layer and the endosperm, and mineral transport routes. Inductive coupled plasma mass spectrometry (ICP‐MS) is a high‐throughput technique used to quantify elements in a vaporised sample (Wilschefski & Baxter, 2019). ICP‐MS has useful for nutritional profiling of wheat grains as it can detect trace amounts of elements (Wu et al., 2013).

### 4.4 Limitations of destructive techniques for grain quality estimation

While destructive techniques discussed above (from section 4.1–4.3) have many uses to study various components of grain, they also provide some challenges. For example, destructive techniques: (1) prevent growth of seed for next generation, which is important for genetic studies; (2) limit secondary analyses of other components, for example, destructive analysis for protein content using Kjeldahl or Dumas would prevent evaluating metabolic profile of the grain or vice versa; (3) are labour‐intensive and expensive, for example, sample preparation or digestion methods require consumables, time and labour each time a set of grains is analysed. In comparison, non‐destructive techniques (for example, hyperspectral imaging) require developing a one‐time calibration that can be used to quantify trait data from future samples; (4) require a higher volume of grain for analysis, for example, Kjeldahl or Dumas require a minimum of 1 g—approximately 15 grains, compared to hyperspectral imaging (discussed later in the review) which can analyse a single grain; and (5) are generally not high throughput due to expensive sample preparation steps (Table 1). Therefore, non‐destructive techniques provide significant benefits to investigate grain quality characteristics.

**TABLE 1:** Advantages and disadvantages of different techniques used for grain quality estimations in food industry and agriculture.

Technique	Advantages	Limitations	High throughput^a^
Microscopy techniques			
TEM	Intracellular structure High‐resolution (down to 0.2 nm)	Laborious sample preparation (dehydration) and fixation Requires thin sample sectioning Expensive set up	No
SEM	Images sample surface High‐resolution (down to <1 nm) Tolerates larger samples compared to TEM	Laborious sample preparation, fixation and metal coating Samples limited to dry phase Expensive set‐up	No
CLSM	Fixation requirement sample dependent Can be non‐destructive Can image tissue sections or small organs Compatible with 3D live imaging	Requires sample staining or fluorescence Lower resolution than electron microscopy	No
AFM	Atomic‐level resolution Measures mechanical properties of sample Cheap operation Requires minimal sample preparation Can be non‐destructive Does not require vacuum to operate	Only analyses a small section of the sample surface	No
X‐ray CT	Fast and non‐destructive Resolution varies, down to nanometre scale Can create 3D scans	Relatively expensive set‐up Requires strong computing power to reconstruct and store 3D images	Yes
Chromatographic techniques			
GelPC	Versatile technique Simple sample preparation	Low‐separation power Sample dilution Long run duration May require large volumes of solvent	No
HPLC	Compatible with many detection methods Elute characterisation possible with MS and NMR Suitable for isolation and quantification	May require derivatisation to improve resolution Requires large amounts of solvent Low sensitivity Does not tolerate volatile substances	Yes
HPAEC	Suitable for isolation and quantification Works well with carbohydrates High sensitivity (pmol) Fast and accurate Compatible with MS	Challenging calibration Sample preparation may be difficult Limited to proteins and carbohydrates Requires high‐pH solvents	No
GC	Suitable for isolation and quantification High sensitivity Fast and accurate Compatible with a range of detection methods, including MS Requires small sample amounts (μL)	Limited to volatile or semi‐volatile compounds Sample must be thermostable	Yes
Spectrometric techniques			
MS	Great to identify substance presence Possible to predict molecular structure High sensitivity (pg) and selectivity Selectivity improved with tandem MS/MS	Expensive set up Sample must ionise well Unreliable separation of similar hydrocarbon ions Unable to distinguish between optical and geometrical isomers Ultimately dependent on the substance purification technique	Yes
NanoSIMS	Elemental and isotopic mapping High‐sensitivity and resolution (50 nm) Compatible with light and electron microscopes	Laborious sample preparation (dehydration) and fixation Expensive set‐up	No
ICP‐MS	High sensitivity Fast and accurate Multiple elements detectable Low sample volumes required Compatible with GC and LC	Expensive set up Environment must be tightly controlled to minimise interference	Yes
ICP‐AES	Simple preparation and low sample volumes required Relatively inexpensive set up	Low sensitivity Limited range of elements detected Limited compatibility with GC and LC	Yes
Non‐destructive spectroscopic techniques			
NMR	Great to elucidate atomic structure High replicability Simple sample preparation Non‐destructive	Expensive set up Solvent used in purification may interfere with measurements Accuracy dependent on the substance purification technique	No
EDS	Compatible with SEM Elemental mapping Multiple elements detectable Can be non‐destructive and require minimal sample preparation	Cannot determine elements below atomic number 11 (sodium) Only provides relative quantification of abundance Unable to distinguish isotopes and ionisation state Sensitivity dependent on compound concentration	No
WDS	Compatible with SEM Elemental mapping Fast and reliable Multiple elements detectable Can be non‐destructive and require minimal sample preparation Improved resolution and sensitivity relative to EDS	Cannot determine elements below atomic number 5 (boron) Only provides relative quantification of abundance Unable to distinguish isotopes and ionisation state Sensitivity dependent on compound concentration	No
NIR	Fast and reliable Non‐destructive Cheap High tissue penetration Requires minimal to no sample preparation	Requires large datasets and tests to build calibration models Lacks specificity (i.e. difficult to characterise at molecular level)	Yes
FT‐IR	Fast and reliable High sensitivity Non‐destructive Requires minimal to no sample preparation	Requires large datasets and tests to build calibration models Requires dehydrated samples Limited to sample surface analysis (packaging is challenging)	Yes
RS	Fast and reliable High sensitivity Non‐destructive Compatible with light microscopes to Achieve cellular resolution Requires minimal to no sample preparation	Sample fluorescence can interfere with detectors Requires optimisation to detect the substance of interest Sample heating from laser may cause destruction	Yes
HSI	Accurate and reliable Cost and labour effective compared to conventional lab techniques Robust Non‐destructive Can study external and internal (partially) structures High resolution Single grain application in cereals Potential for plant breeding	Produces large amounts of data Chemometrics is a challenge Requires strong computing power and high‐performance systems Skills from many fields required Higher noise compared to bench NIR systems	Yes

Abbreviations: AFM, atomic force microscopy; CLSM, confocal laser scanning microscopy; EDS, energy‐dispersive X‐ray spectroscopy; FT‐IR, Fourier‐transform infrared spectroscopy; GC, gas chromatography; GelPC, gel permeation chromatography; HPAEC, high‐performance anion exchange chromatography; HPLC, high‐performance liquid chromatography; HSI, hyperspectral imaging; ICP‐AES, inductively coupled plasma atomic emission spectrometry; ICP‐MS, inductively coupled plasma mass spectrometry; MS, mass spectroscopy; NanoSIMS, nanoscale secondary ion mass spectrometry; NIR, near‐infrared spectroscopy; NMR, nuclear magnetic resonance; RS, Raman spectroscopy; SEM, scanning electron microscopy; TEM, transmission electron microscopy; WDS, wavelength‐dispersive X‐ray spectroscopy; X‐ray CT, X‐ray computed tomography.

^a^ ‘Yes’ means it can be high throughput depending on computational setup and application.

### 4.5 Non‐destructive spectroscopic techniques

Spectroscopic techniques are used in food science for the quality assessment of grains and to evaluate their chemical composition. Some commonly used non‐destructive spectroscopic methods are briefly discussed here. Energy dispersive spectroscopy (EDS) is used to complement SEM. It is performed rapidly, and its sensitivity is limited to the concentration of compounds and provides atomic information (Ngo, 1999). It has been used in wheat to determine nutrient concentrations for example, Fe, Zn and Se in grains (Paltridge et al., 2012), and Ca, K, P, Mg, Se, Cu, S, Mn, Fe and Zn in flour (Peruchi et al., 2014). Wavelength dispersive spectroscopy (WDS) is similar to EDS except that it provides better resolution and prevents overlaps in peak areas that occur commonly with EDS. Nuclear magnetic resonance (NMR) is used to determine the structures and compositions of novel and previously known chemical compounds. NMR applications in food science include investigating the differences in flour quality (Tsirivakou et al., 2020), chemical and genetic diversity of polar metabolites (Shewry et al., 2017), and studying changes in grain components (Poudel et al., 2021). It is important to note that while NMR itself is considered a non‐destructive technique, the sample preparation can sometimes be invasive, depending on the nature of the sample.

Near‐Infrared (NIR) spectroscopy is heavily reliant on statistical analysis to correlate the NIR spectra and the target compound. NIR can be used for several purposes such as measuring protein and nitrogen levels, detecting disease, managing plant health and monitoring grain development (Su et al., 2017). The application of NIR for wheat quality assessment started many years ago (Biston, 1982; Downey & Byrne, 1983), and has become an established technique for quality assessment of foods in the cereal‐processing industry (Caporaso et al., 2018b). Fourier transform infrared (FT‐IR) spectroscopy is more sensitive than NIR because it measures the fundamental vibrations of functional group bonds, while NIR detects the wave harmonics. However, FT‐IR does not penetrate as deep in tissue samples nor tolerate water. The technique is gaining popularity in food quality assessment (Ellis et al., 2012), for example, to detect wheat flour adulteration with barley flour (Arslan et al., 2020). Raman spectroscopy (RS) works beyond the infra‐red spectrum, up to ultra‐violet so, unlike FT‐IR and NIR, it can be used through transparent materials (packaging) and has minimal interference from water. The technique is fast, simple, can detect a wide range of analytes, and can be paired with light microscopes, enabling single‐cell resolution. Like other spectroscopy techniques, it is used in food quality assessment, for example: to measure flour purity (Czaja et al., 2020) and gluten proteins quantification (Czaja et al., 2016).

While spectroscopic techniques have many advantages for food quality assessment, there are some limitations. For instance, EDS and WDS are limited in sensitivity to the concentration of compounds and atomic information. NMR is limited in its ability to analyse small molecules and can be challenging to use for quantification. NIR spectroscopy relies heavily on statistical analysis and requires bigger sample size, while RS can suffer from fluorescence interference, and its signal‐to‐noise ratio can be lower compared to other techniques. Additionally, the application of these techniques is not extended to phenotyping large plant populations in field trials. Table 1 presents the benefits and limitations of the different methods discussed here. In the next section, we will discuss how hyperspectral imaging (HSI, hereafter) stands out from other techniques for the quality assessment of wheat grain and its application in plant breeding.

## 5 HSI—AN INNOVATIVE TECHNOLOGY FOR STUDYING GRAIN QUALITY TRAITS

HSI is an innovative and powerful imaging technique that combines NIR with a broad‐spectrum camera to analyse the spatial distribution of food quality parameters. Unlike traditional imaging techniques, HSI allows for non‐destructive and rapid analysis of large‐scale field trials, as well as single grain analysis. It is rapidly becoming the preferred method for food quality assessment in industry. Compared to other techniques, HSI stands out due to its ability to capture a vast amount of data from the NIR spectrum, resulting in higher resolution and sensitivity. This enables HSI to identify subtle changes in food quality parameters, such as moisture content, texture and protein content, that may not be detectable by other imaging techniques. Additionally, HSI can provide a more comprehensive and holistic view of the food sample, enabling the identification of both surface and internal defects. HSI surpasses other techniques in terms of accuracy, speed and non‐destructiveness, making it the ideal choice for food quality assessment and largescale phenotyping for grain quality traits. In the following section, we will focus on a HSI system, data acquisition, data processing and analysis, and the application of HSI for determining wheat grain quality traits.

**FIGURE 4:**
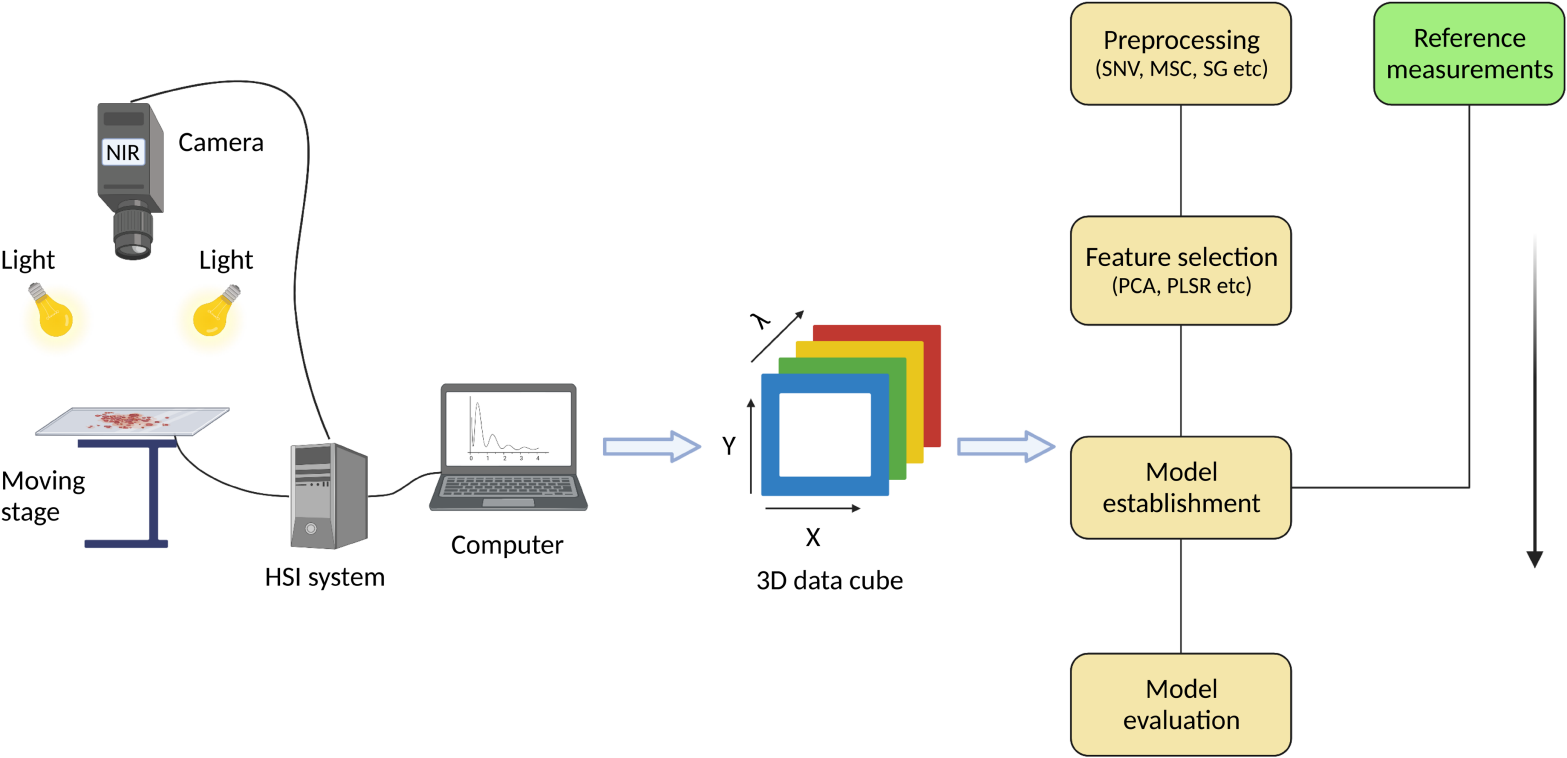
A schematic diagram of hyperspectral imaging and data analysis. A typical indoor hyperspectral system consists of a movable stage for sample placement, a light source with equal scattering, a near‐infrared camera, a hyperspectral system that connects the stage and the camera and a computer interface to collect data. Data are produced as 3D hypercubes which are processed using chemometrics to extract desired information. A reference material is used to build a calibration for the quantification of trait data from future samples using only hyperspectral data (Created with BioRender.com).

There are commonly three kinds of hyperspectral systems known as whiskbroom, pushbroom and tuneable filter which vary slightly in terms of their application in different industries (Elmasry et al., 2012). The most used system in food industry is the pushbroom system. A typical pushbroom system consists of a broad spectral camera system, a spectrograph with a c‐mount lens, a moving stage to place the samples, an illumination unit and a computer interface to produce images and data. The camera detects in two dimensions to collect both spatial and spectral information, and the spectrograph generates a spectrum for each point on the scanned line. Hence, a three‐dimensional hyperspectral image called ‘hypercube’ is generated by scanning the entire surface of an object (Figure 4).

### 5.1 Hyperspectral data acquisition and processing

Biological systems absorb or emit energy when struck by electromagnetic radiation (light). HSI data is the absorbed or emitted energy by a biological system under screening stored in the form of a 3D image, the hypercube (Ravikanth et al., 2017). The hypercube is a 3D cube produced from hundreds of wavelength bands at each pixel of an image detected by the HSI sensor. The commonly used pushbroom line scanner HSI sensor produces all wavelength bands along the same spatial coordinate as the line direction (Lawrence et al., 2003), resulting in a 3D hypercube in either of the three ENVI (the Environment for Visualising Images) formats that is, BIL (band interleaved by line), BSQ (band sequential) and BIP (band interleaved by pixel). The selection of optimal format is recommended before proceeding to the data analysis. Generally, the BIL format provides a good account of image processing tasks and is used commonly. A complete scheme of HSI data analysis is reviewed elsewhere (Yoon & Park, 2015). A brief description is given below and in Figure 4. It is important to note the illustration demonstrates the ability of indoor HSI systems to examine grain quality features. Fortunately, a recent review has demonstrated the application of outdoor HSI systems in phenotyping plant traits (Sarić et al., 2022).

Calibration of the data is required to ensure the accuracy and reproducibility of the results. Calibration can be of three types: (a) spectral calibration where wavelengths are linked with band numbers, (b) spatial calibration where each image pixel is correlated to a known feature and (c) radiometric calibration which in food technology simply refers to reflectance (or transmittance) calibration (Yoon & Park, 2015). Care must be taken when collecting data for reference spectra from field, because the dynamic environment in which crops grow can impact the hyperspectral calibration, such as through wind speed, cloud cover, light intensity and angle, air pressure, and temperature and humidity (Pfitzner et al., 2011). The first step in image processing is binarization (also called thresholding) by which a binary image is produced by masking the image background (Yoon et al., 2009). A systematic approach for background masking such as principal component analysis can also be used to select the band with the largest reflectance variation. After masking, the foreground picture elements are used to create the region of interest (ROI), which includes the area of image used to extract the spectral information (Yoon & Park, 2015). Spectral pre‐processing algorithms are applied to select the optimal wavelength to account for confounding factors such as random noise, light scattering and variation in the length of the light path (Ma et al., 2019). Principal component analysis (PCA) is the most common unsupervised method to reduce the dimension of hypercube and eliminate correlated wavelengths (Minaei et al., 2017). For model development, two types of chemometric models that is, classification and quantification models are used.

Classification models can be either supervised, when a reference value or class is available, or unsupervised, when reference values are unavailable. Examples of supervised classification models include artificial neural networks (ANN), support vector machine (SVM), decision trees (DT), random forest (RF), k‐nearest neighbour (k‐NN), logistic regression (LR), naive bayes (NB) and linear discriminant analysis (LDA). Among the unsupervised classification models, K‐means clustering (KMC) and PCA are most frequently used followed by independent component analysis (ICA) (Jiang et al., 2010).

Quantification models are used to establish relationship between the hyperspectral data and the desired attributes to provide a continuous numerical prediction. Examples include regression models such as multiple linear regression (MLR), partial least squares regression (PLSR) and principal component regression (PCR) (Pan et al., 2016). Many computer applications and algorithms have been developed such as MATLAB image processing toolbox and PlantCV in Python (Gehan et al., 2017) to process and analyse the hyperspectral image data. Analyses depend on the type of questions asked during the experiment.

### 5.2 Application of HSI to study grain quality traits in wheat

Grain quality in wheat, as discussed earlier, is determined by several properties such as hardness, moisture content, nitrogen and protein content, insect damage and falling number. HSI has developed into a powerful tool to determine these quality indicators in wheat industry. In a study on insect damaged wheat from Canada, the authors classified healthy and damaged grains successfully by applying multivariate regression model on hyperspectral data (Singh et al., 2009). In other studies, HSI was used to study nitrogen content (Vigneau et al., 2011), fungal infection (Singh et al., 2007), diffusion of water in different hardness levels (Manley et al., 2011) and protein content (Caporaso et al., 2018a).

Among the different quality attributes, protein content and composition have major influence on wheat grain quality which are both affected by nitrogen level during plant development. The reflectance spectrum of electromagnetic waves can be affected by the chlorophyl pigment especially in blue (450 nm) and red (670 nm) bands in wheat plants which is related to leaf nitrogen content (Gamon et al., 1997; Le Maire et al., 2004). Recently, significant progress has been made in the field of reflectance spectral analysis of different vegetation indices (VIs), including normalised difference vegetation index (NDVI) (Hansen & Schjoerring, 2003), medium terrestrial chlorophyll index (MTCI) and normalised pigments chlorophyll ratio index (NPCRI) (Tan et al., 2018), normalised water index (NWI) (Babar et al., 2006) and structural insensitive pigment index (SIPI) (Robles‐Zazueta et al., 2021) which are derived from the canopy with respect to plant nitrogen content. HSI application using different VIs to estimate plant nitrogen content has been reviewed elsewhere (Ma et al., 2022).

Nowadays, satellite spectral images provide freely available data source for wheat nutrition and grain quality monitoring. An example can be seen in a previous study where authors assessed the ability of spectral VIs of Sentinel‐2 data (Sentinel‐2 is an earth observation mission to monitor land and sea, sea ice and natural disasters) for the detection of nitrogen and GPC based on different VIs (Zhao et al., 2019). Recently, HSI was successfully used to predict micronutrients in wheat (Hu et al., 2021). These recent advances and successes demonstrate the ability of HSI to study grain quality traits in wheat and potential to transform the technique into largescale phenotyping trials. Table 2 provides an account of recent studies using HSI to investigate wheat quality parameters.

**TABLE 2:** Various applications of hyperspectral imaging for evaluating different quality parameters in wheat including grain protein content.

Product	Application	Data analysis method	Wavelength (nm)	References
Single grain	Predicting carbon and nitrogen concentrations	Partial least square regression (PLSR)	1000–2500	Tahmasbian et al. (2021)
Grain and flour	Predicting micronutrients in wheat	PLSR	375–1050	Hu et al. (2021)
Whole plant	Predicting yield and biomass	PLSR	350–2500	Robles‐Zazueta et al. (2021)
Whole plant	Predicting protein content	Linear regression, machine learning	530–810	Zhou, Kono, et al. (2021)
Whole (ground)	Determining intestinal crude protein digestibility	PLSR	680–2500	Shi et al. (2019)
Wheat gluten	Detecting gluten content	Wavelet soft‐threshold method	1300–2300	Cai (2017)
Whole plant	Predicting yield and biomass	Linear regression	350–2500	Tan et al. (2018)
Single grain	Predicting protein variation	PLSR, PCA	980–2500	Caporaso et al. (2018a)
Bulk grains	Classifying vitreous/non vitreous kernels	Savitzky–Golay, first derivative	950–2450	Shahin and Symons (2008)
Bulk grains	Classifying eight different Canadian wheats	Linear‐ and quadratic‐discriminant analysis	960–1700	Mahesh et al. (2008)
Single grain	Classifying sound/stained grain	Multivariate image analysis (MVIA)	350–2500	Berman et al. (2007)
Single grain	Detecting fungal infection	MVIA based on PCA	1000–1600	Singh et al. (2007)
Whole plant	Quantifying stripe rust disease index	Photochemical reflectance index (PRI)	1050–2500	Huang et al. (2007)
Whole plant	Predicting yield and biomass	PLSR	350–2500	Babar et al. (2006)
Whole plant	Predicting leaf nitrogen content	PLSR	438–884	Hansen and Schjoerring (2003)

### 5.3 HSI for studying phenotypic variation at single grain level

HSI can extract information rapidly and non‐destructively from single grains without the need of grinding them into powder. Grinding material provides more uniform accuracy but is a time consuming and laborious effort and removes the intra‐sample variability across single grains. Therefore, application of HSI on whole single grains has gained a lot of interest in the recent years. NIR spectroscopy was first applied to study protein content from single grains in 1995 to study the grain protein content by transmittance method (Delwiche, 1995). In a recent study, the diffusion of conditioning water was studied by HSI in single wheat grains of different hardness levels. From the hyperspectral data, the authors were able to predict that the uptake of water followed a pattern from soft toward hard grains. They also observed that the protein content was higher in hard grains (Manley et al., 2011). In another study of four different wheat classes from USA, protein variability was successfully determined from single grains in more than 300 samples by using NIR spectroscopy (Delwiche, 1998). A more recent study estimated single grain protein content in 180 wheat cultivars with 10 seeds from each sample totalling prediction for 4200 single wheat grains using HIS (Caporaso et al., 2018a). These studies indicate that HSI can be a powerful and reliable tool to robustly and non‐destructively phenotype single grains in large populations.

### 5.4 Recent developments and prospects on the application of HSI in plant breeding

HSI provides extensive information that correlates well with chemical constituents, additives and mycotoxin; it has already been widely used to measure food parameters, including those related to protein and nitrogen content. Because HSI is massively high throughput compared to some of the other commonly used methods, it can be suitable for largescale phenotyping in plant breeding. Plant traits predicted using HSI have been used in genome‐wide association studies to identify novel candidate genes, including those related to yield in wheat (Fei et al., 2022) and grain quality in rice (Sun et al., 2019). Application of HSI in plant phenotyping has extended to crop yield, quality, stress response, architecture and root morphology in both controlled and natural environments (reviewed recently by Sarić et al. (2022)). A relatively unexplored area has been the ability of HSI to analyse variation at single grain level that provides major benefits for studying grain heterogeneity or homogeneity traits. Variation for grain quality traits such as protein content at single grain level across a population could help rapidly pinpoint genetic basis controlling these traits towards providing genetic solutions for breeding varieties with homogeneity for grain quality traits. Additionally, assessment of grain protein at single grain level non‐destructively can allow selection for grain protein at early generations in breeding programme, when only small number of seed is available per progeny and the seed of selected lines is required for sowing the next generation.

However, due to the large amount of data generated in hyperspectral images and the difficulty to detect molecular features of the compounds, chemometric modelling remains a challenge. It can be time consuming at times to build the right model, and expertise from different fields such as chemistry and computer science may need to be integrated to solve the problem. Another limitation of HSI is to identify unique variation in the test set that could be outside the range of the calibration model, for example, in plant breeding research, genetic variation originating from wild resources can sometime have a large effect on a phenotype, whereas a hyperspectral system will rely on a certain range of the calibration dataset for prediction. Therefore, for research on complex plant traits and germplasms having historically and geographically wider distribution, a separate calibration set might be required each time or one with enough diversity to cover broad variation. The dataset must also be large and representative of the whole population under study. The major challenge, however, remains the chemometrics and data analysis, including the speed of analysis needed for full industrial applications. Because the data generated by hyperspectral systems consists of a continuous series of wavelength bands and a large number of pixels belonging to the same object, many image cleaning and spectral pre‐processing steps are required to obtain any useful information, thus involving complex mathematical operations requiring strong computational capacity (Yoon & Park, 2015). This provides a potential area of improvement in HSI in future research by taking advantage of emerging software technology and machine learning algorithms. Introduction of automated models for large datasets and user‐friendly graphical user interfaces compared to the algorithms run by coding languages will be desirable outcomes for breeders and plant biologists and will also extend the application of the technology.

While chemometrics has advanced significantly in recent years, there are still challenges to be addressed, for example, regarding the use of unlabelled data in unsupervised models. Additionally, intelligent recognition systems and fully automatic and customisable models are still under development. Another challenge is the chemical analysis of small sample sizes, such as the characterisation of single grain as current analytical methods require a minimum sample size larger than single grains, for example, Kjeldahl or Dumas require ~15 wheat grains for analysis. Overcoming these challenges is critical for advancing the field of HSI and unlocking its full potential in various applications, such as food quality control and breeding for grain quality.

## 6 CONCLUSION

Our study discusses how a focus on yield during modern breeding could have resulted in allelic loss for grain quality traits in wheat. It further discusses the challenge faced by plant breeders to simultaneously phenotype grain yield and quality traits with traditional methods and the advantage of HSI that could be exploited to revive grain quality. HSI can rapidly and non‐destructively phenotype key traits controlling grain quality at single grain levels. Future research can benefit from accelerated phenotyping through HSI at single grain levels to expedite genetic studies for the identification of novel alleles underlying homogeneity and heterogeneity for grain quality traits.

## FUNDING INFORMATION

This work was supported by the Biotechnology and Biological Sciences Research Council (grant numbers BB/V018108/1, BB/W006979/1, BB/V004115/1); University of Adelaide Research Scholarship; and the Australian Research Council (FT210100810).

## CONFLICT OF INTEREST STATEMENT

The authors declare no competing interests.

## Data Availability

Data sharing is not applicable to this article as no new data were created or analyzed in this study.

## References

[fes3498-bib-0001] Ahmed, Z. A. , Nadulski, R. , Kobus, Z. , & Zawiślak, K. (2015). The influence of grain moisture content on specific energy during spring wheat grinding. Agriculture and Agricultural Science Procedia, 7, 309–312.

[fes3498-bib-0002] Andersson, A. A. , Andersson, R. , Piironen, V. , Lampi, A.‐M. , Nyström, L. , Boros, D. , Fraś, A. , Gebruers, K. , Courtin, C. M. , & Delcour, J. A. (2013). Contents of dietary fibre components and their relation to associated bioactive components in whole grain wheat samples from the HEALTHGRAIN diversity screen. Food Chemistry, 136, 1243–1248. 23194520 10.1016/j.foodchem.2012.09.074

[fes3498-bib-0003] Arslan, F. N. , Akin, G. , Karuk Elmas, Ş. N. , Üner, B. , Yilmaz, I. , Janssen, H.‐G. , & Kenar, A. (2020). FT‐IR spectroscopy with chemometrics for rapid detection of wheat flour adulteration with barley flour. Journal of Consumer Protection and Food Safety, 15, 245–261. 10.1007/s00003-019-01267-9

[fes3498-bib-0004] Babar, M. , Reynolds, M. , Van Ginkel, M. , Klatt, A. , Raun, W. , & Stone, M. (2006). Spectral reflectance indices as a potential indirect selection criteria for wheat yield under irrigation. Crop Science, 46, 578–588.

[fes3498-bib-0005] Barnard, A. D. , Labuschagne, M. T. , & van Niekerk, H. A. (2002). Heritability estimates of bread wheat quality traits in the Western cape province of South Africa. Euphytica, 127, 115–122. 10.1023/A:1019997427305

[fes3498-bib-0006] Berman, M. , Connor, P. , Whitbourn, L. , Coward, D. , Osborne, B. , & Southan, M. (2007). Classification of sound and stained wheat grains using visible and near infrared hyperspectral image analysis. Journal of Near Infrared Spectroscopy, 15, 351–358.

[fes3498-bib-0007] Biesiekierski, J. R. (2017). What is gluten? Journal of Gastroenterology and Hepatology, 32, 78–81. 28244676 10.1111/jgh.13703

[fes3498-bib-0008] Biston, R. & Clamot, G. (1982). Use of near‐infrared reflectance spectroscopy and dye‐binding techniques for estimating protein in oat groats. Cereal Chemistry, 59(5), 333–335.

[fes3498-bib-0009] Bleukx, W. , Torrekens, S. , Van Leuven, F. , & Delcour, J. (1998). Purification, properties and N‐terminal amino acid sequence of a wheat gluten aspartic proteinase. Journal of Cereal Science, 28, 223–232.

[fes3498-bib-0010] Bogard, M. , Allard, V. , Brancourt‐Hulmel, M. , Heumez, E. , Machet, J.‐M. , Jeuffroy, M.‐H. , Gate, P. , Martre, P. , & Le Gouis, J. (2010). Deviation from the grain protein concentration–grain yield negative relationship is highly correlated to post‐anthesis N uptake in winter wheat. Journal of Experimental Botany, 61, 4303–4312. 10.1093/jxb/erq238 20679251

[fes3498-bib-0011] Brinton, J. , & Uauy, C. (2019). A reductionist approach to dissecting grain weight and yield in wheat. Journal of Integrative Plant Biology, 61, 337–358. 30421518 10.1111/jipb.12741 PMC6492019

[fes3498-bib-0012] Cai, J.‐H. (2017). Near‐infrared spectrum detection of wheat gluten protein content based on a combined filtering method. Journal of AOAC International, 100, 1565–1568. 28425394 10.5740/jaoacint.17-0008

[fes3498-bib-0013] Caporaso, N. , Whitworth, M. B. , & Fisk, I. D. (2017). Application of calibrations to hyperspectral images of food grains: Example for wheat falling number. Journal of Spectral Imaging, 6, 1–15.

[fes3498-bib-0014] Caporaso, N. , Whitworth, M. B. , & Fisk, I. D. (2018a). Protein content prediction in single wheat kernels using hyperspectral imaging. Food Chemistry, 240, 32–42. 28946278 10.1016/j.foodchem.2017.07.048 PMC5625851

[fes3498-bib-0015] Caporaso, N. , Whitworth, M. B. , & Fisk, I. D. (2018b). Near‐infrared spectroscopy and hyperspectral imaging for non‐destructive quality assessment of cereal grains. Applied Spectroscopy Reviews, 53, 667–687.

[fes3498-bib-0016] Carbonero, P. , & García‐Olmedo, F. (1999). A multigene family of trypsin/α‐amylase inhibitors from cereals. In P. R. Shewry & R. Casey (Eds.), Seed proteins (pp. 617–633). Springer.

[fes3498-bib-0017] Cauvain, S. P. (2012). Breadmaking: Improving quality. Elsevier.

[fes3498-bib-0018] Chichti, E. , George, M. , Delenne, J.‐Y. , & Lullien‐Pellerin, V. (2015). Changes in the starch‐protein interface depending on common wheat grain hardness revealed using atomic force microscopy. Plant Science, 239, 1–8. 26398785 10.1016/j.plantsci.2015.07.006

[fes3498-bib-0019] Chung, O. (1986). Lipid‐protein interactions in wheat flour, dough, gluten, and protein fractions. Cereal Foods World (USA).

[fes3498-bib-0020] Clarke, M. P. , Gooding, M. J. , & Jones, S. A. (2004). The effects of irrigation, nitrogen fertilizer and grain size on Hagberg falling number, specific weight and blackpoint of winter wheat. Journal of the Science of Food and Agriculture, 84, 227–236.

[fes3498-bib-0021] Cleemput, G. , Hessing, M. , van Oort, M. , Deconynck, M. , & Delcour, J. A. (1997). Purification and characterization of a [beta]‐D‐xylosidase and an endo‐xylanase from wheat flour. Plant Physiology, 113, 377–386. 12223612 10.1104/pp.113.2.377 PMC158151

[fes3498-bib-0022] Conner, R. , & Kuzyk, A. (1988). Effectiveness of fungicides in controlling stripe rust, leaf rust, and black point in soft white spring wheat. Canadian Journal of Plant Pathology, 10, 321–326.

[fes3498-bib-0023] Czaja, T. , Mazurek, S. , & Szostak, R. (2016). Quantification of gluten in wheat flour by FT‐Raman spectroscopy. Food Chemistry, 211, 560–563. 10.1016/j.foodchem.2016.05.108 27283667

[fes3498-bib-0024] Czaja, T. , Sobota, A. , & Szostak, R. (2020). Quantification of ash and moisture in wheat flour by Raman spectroscopy. Food, 9, 280. 10.3390/foods9030280 PMC7143060 32138384

[fes3498-bib-0025] Debyser, W. , & Delcour, J. (2008). Inhibitors of cellulolytic, xylanolytic and β‐glucanolytic enzymes . U.S. Patent 7, 393, 929. 1 Jul. 2008.

[fes3498-bib-0026] Delwiche, S. (1998). Protein content of single kernels of wheat by near‐infrared reflectance spectroscopy. Journal of Cereal Science, 27, 241–254.

[fes3498-bib-0027] Delwiche, S. R. (1995). Single wheat kernel analysis by near‐infrared transmittance: Protein content. Cereal Chemistry, 72(1), 11‐16.

[fes3498-bib-0028] Distelfeld, A. , Uauy, C. , Olmos, S. , Schlatter, A. R. , Dubcovsky, J. , & Fahima, T. (2004). Microcolinearity between a 2‐cM region encompassing the grain protein content locus Gpc‐6B1 on wheat chromosome 6B and a 350‐kb region on rice chromosome 2. Functional & Integrative Genomics, 4, 59–66. 14752608 10.1007/s10142-003-0097-3

[fes3498-bib-0029] Downey, G. , & Byrne, S. (1983). Determination of protein and moisture in ground wheat by near infra‐red reflectance spectroscopy. Irish Journal of Food Science and Technology, 7, 135–146.

[fes3498-bib-0030] Dziki, D. , & Przypek‐Ochab, D. (2009). Ocena energochłonności rozdrabniania ziarna pszenicy zróżnicowanego pod względem twardości. Inżynieria Rolnicza, 13, 61–67.

[fes3498-bib-0031] Ellis, D. I. , Brewster, V. L. , Dunn, W. B. , Allwood, J. W. , Golovanov, A. P. , & Goodacre, R. (2012). Fingerprinting food: Current technologies for the detection of food adulteration and contamination. Chemical Society Reviews, 41, 5706–5727. 10.1039/C2CS35138B 22729179

[fes3498-bib-0032] Elmasry, G. , Kamruzzaman, M. , Sun, D.‐W. , & Allen, P. (2012). Principles and applications of hyperspectral imaging in quality evaluation of Agro‐food products: A review. Critical Reviews in Food Science and Nutrition, 52, 999–1023. 10.1080/10408398.2010.543495 22823348

[fes3498-bib-0033] Enghiad, A. , Ufer, D. , Countryman, A. M. , & Thilmany, D. D. (2017). An overview of global wheat market fundamentals in an era of climate concerns. International Journal of Agronomy, 2017, 3931897.

[fes3498-bib-0034] Evers, A. (2000). Grain size and morphology: Implications for quality. In D. Schofield (Ed.), Wheat structure, biochemistry and functionality. Royal Society of Chemistry.

[fes3498-bib-0035] FAO . (2022). Statistics Database for Crops . License: CC BY‐NC‐SA 3.0 IGO. https://www.fao.org/faostat/en/?#data/QCL. Accessed September 1, 2022.

[fes3498-bib-0036] Fei, S. , Hassan, M. A. , Xiao, Y. , Rasheed, A. , Xia, X. , Ma, Y. , Fu, L. , Chen, Z. , & He, Z. (2022). Application of multi‐layer neural network and hyperspectral reflectance in genome‐wide association study for grain yield in bread wheat. Field Crops Research, 289, 108730.

[fes3498-bib-0037] Foulkes, M. J. , Hawkesford, M. J. , Barraclough, P. B. , Holdsworth, M. J. , Kerr, S. , Kightley, S. , & Shewry, P. R. (2009). Identifying traits to improve the nitrogen economy of wheat: Recent advances and future prospects. Field Crops Research, 114, 329–342. 10.1016/j.fcr.2009.09.005

[fes3498-bib-0038] Fra‐Mon, P. , Salcedo, G. , Aragoncillo, C. , & Garcia‐Olmedo, F. (1984). Chromosomal assignment of genes controlling salt‐soluble proteins (albumins and globulins) in wheat and related species. Theoretical and Applied Genetics, 69, 167–172. 24253707 10.1007/BF00272890

[fes3498-bib-0039] Gamon, J. , Serrano, L. , & Surfus, J. (1997). The photochemical reflectance index: An optical indicator of photosynthetic radiation use efficiency across species, functional types, and nutrient levels. Oecologia, 112, 492–501. 28307626 10.1007/s004420050337

[fes3498-bib-0040] Gao, S. , Gu, Y. Q. , Wu, J. , Coleman‐Derr, D. , Huo, N. , Crossman, C. , Jia, J. , Zuo, Q. , Ren, Z. , & Anderson, O. D. (2007). Rapid evolution and complex structural organization in genomic regions harboring multiple prolamin genes in the polyploid wheat genome. Plant Molecular Biology, 65, 189–203. 17629796 10.1007/s11103-007-9208-1

[fes3498-bib-0041] Gaurav, K. , Arora, S. , Silva, P. , Sánchez‐Martín, J. , Horsnell, R. , Gao, L. , Brar, G. S. , Widrig, V. , John Raupp, W. , Singh, N. , Wu, S. , Kale, S. M. , Chinoy, C. , Nicholson, P. , Quiroz‐Chávez, J. , Simmonds, J. , Hayta, S. , Smedley, M. A. , Harwood, W. , … Wulff, B. B. H. (2022). Population genomic analysis of *Aegilops tauschii* identifies targets for bread wheat improvement. Nature Biotechnology, 40, 422–431. 10.1038/s41587-021-01058-4 PMC8926922 34725503

[fes3498-bib-0042] Gedye, D. J. , Doling, D. A. , & Kingswood, K. W. (1981). A farmers guide to wheat quality. National Agricultural Centre, Cereal Unit.

[fes3498-bib-0043] Gegas, V. C. , Nazari, A. , Griffiths, S. , Simmonds, J. , Fish, L. , Orford, S. , Sayers, L. , Doonan, J. H. , & Snape, J. W. (2010). A genetic framework for grain size and shape variation in wheat. The Plant Cell, 22, 1046–1056. 20363770 10.1105/tpc.110.074153 PMC2879751

[fes3498-bib-0044] Gehan, M. A. , Fahlgren, N. , Abbasi, A. , Berry, J. C. , Callen, S. T. , Chavez, L. , Doust, A. N. , Feldman, M. J. , Gilbert, K. B. , Hodge, J. G. , Hoyer, J. S. , Lin, A. , Liu, S. , Lizárraga, C. , Lorence, A. , Miller, M. , Platon, E. , Tessman, M. , & Sax, T. (2017). PlantCV v2: Image analysis software for high‐throughput plant phenotyping. PeerJ, 5, e4088. 10.7717/peerj.4088 29209576 PMC5713628

[fes3498-bib-0045] Giles, J. (2005). Nitrogen study fertilizes fears of pollution. Nature, 433, 791. 15729306 10.1038/433791a

[fes3498-bib-0046] GEF Secretariat . (2023). Combating Land Degradation. Global Environment Facility. https://www.thegef.org/newsroom/publications/combating‐land‐degradation. Accessed May 15, 2023.

[fes3498-bib-0047] Goesaert, H. , Gebruers, K. , Courtin, C. , Brijs, K. , & Delcour, J. (2006). Enzymes in breadmaking. In Y. H. Hui (Ed.), Bakery products: Science and technology (pp. 337–364). Wiley.

[fes3498-bib-0048] Grosch, W. , & Wieser, H. (1999). Redox reactions in wheat dough as affected by ascorbic acid. Journal of Cereal Science, 29, 1–16.

[fes3498-bib-0049] Hagberg, S. (1960). A rapid method for determining alpha‐amylase activity. Cereal Chemistry, 37, 218–222.

[fes3498-bib-0050] Hailu, F. , Labuschagne, M. , Van Biljon, A. , Persson Hovmalm, H. , & Johansson, E. (2016). Quality assessment with HPLC in released varieties of tetraploid (*Triticum durum* Desf.) wheat from Ethiopia and Spain. Cereal Research Communications, 44, 617–627.

[fes3498-bib-0051] Halverson, J. , & Zeleny, L. (1988). Criteria of wheat quality. In Y. Pomeranz (Ed.), Wheat: Chemistry and technology (Vol. I, pp. 15–45). AACCI.

[fes3498-bib-0052] Hansen, P. , & Schjoerring, J. (2003). Reflectance measurement of canopy biomass and nitrogen status in wheat crops using normalized difference vegetation indices and partial least squares regression. Remote Sensing of Environment, 86, 542–553.

[fes3498-bib-0053] Hareland, G. A. (2003). Effects of pearling on falling number and α‐amylase activity of preharvest sprouted spring wheat. Cereal Chemistry, 80, 232–237.

[fes3498-bib-0054] Hawkins, E. , Chen, J. , Watson‐Lazowski, A. , Ahn‐Jarvis, J. , Barclay, J. E. , Fahy, B. , Hartley, M. , Warren, F. J. , & Seung, D. (2021). STARCH SYNTHASE 4 is required for normal starch granule initiation in amyloplasts of wheat endosperm. *bioRxiv*. 10.1101/2021.01.29.428798 33714222

[fes3498-bib-0055] Hoseney, R. , Finney, K. , Pomeranz, Y. , & Shogren, M. (1969). Functional (breadmaking) and biochemical properties of wheat flour components. V. Role of total extractable lipids. Cereal Chemistry, 46, 606–613.

[fes3498-bib-0056] Hoseney, R. C. (1994). Principles of cereal science and technology. American Association of Cereal Chemists (AACC).

[fes3498-bib-0057] Hu, N. , Li, W. , Du, C. , Zhang, Z. , Gao, Y. , Sun, Z. , Yang, L. , Yu, K. , Zhang, Y. , & Wang, Z. (2021). Predicting micronutrients of wheat using hyperspectral imaging. Food Chemistry, 343, 128473. 33160768 10.1016/j.foodchem.2020.128473

[fes3498-bib-0058] Huang, W. , Lamb, D. W. , Niu, Z. , Zhang, Y. , Liu, L. , & Wang, J. (2007). Identification of yellow rust in wheat using in‐situ spectral reflectance measurements and airborne hyperspectral imaging. Precision Agriculture, 8, 187–197.

[fes3498-bib-0059] Humphreys, K. J. , Conlon, M. A. , Young, G. P. , Topping, D. L. , Hu, Y. , Winter, J. M. , Bird, A. R. , Cobiac, L. , Kennedy, N. A. , & Michael, M. Z. (2014). Dietary manipulation of oncogenic microRNA expression in human rectal mucosa: A randomized trial. Cancer Prevention Research, 7, 786–795. 25092886 10.1158/1940-6207.CAPR-14-0053

[fes3498-bib-0060] Jacobs, B. , & Rabie, C. (1987). The correlation between mycelial presence and black‐point in barley. Phytophylactica, 19, 77–82.

[fes3498-bib-0061] Jiang, L. , Zhu, B. , & Tao, Y. (2010). CHAPTER 3 – Hyperspectral image classification methods. In D.‐W. Sun (Ed.), Hyperspectral imaging for food quality analysis and control (pp. 79–98). Academic Press.

[fes3498-bib-0062] Kandra, L. (2003). α‐Amylases of medical and industrial importance. Journal of Molecular Structure: THEOCHEM, 666, 487–498.

[fes3498-bib-0063] Karmakar, P. , Murshed, S. W. T. , Pang, P. , & Van Bui, C. (2022). A guide to employ hyperspectral imaging for assessing wheat quality at different stages of supply chain in Australia: A review. *arXiv*. https://arxiv.org/abs/2209.05727

[fes3498-bib-0064] Kim, J. H. , Maeda, T. , & Morita, N. (2006). Effect of fungal α‐amylase on the dough properties and bread quality of wheat flour substituted with polished flours. Food Research International, 39, 117–126.

[fes3498-bib-0065] Kumar, J. , Schäfer, P. , Hückelhoven, R. , Langen, G. , Baltruschat, H. , Stein, E. , Nagarajan, S. , & Kogel, K. H. (2002). Bipolaris sorokiniana, a cereal pathogen of global concern: Cytological and molecular approaches towards better control. Molecular Plant Pathology, 3, 185–195. 20569326 10.1046/j.1364-3703.2002.00120.x

[fes3498-bib-0066] Lawrence, K. C. , Park, B. , Windham, W. R. , & Mao, C. (2003). Calibration of a pushbroom hyperspectral imaging system for agricultural inspection. Transactions of ASAE, 46, 513.

[fes3498-bib-0067] Le Maire, G. , François, C. , & Dufrene, E. (2004). Towards universal broad leaf chlorophyll indices using PROSPECT simulated database and hyperspectral reflectance measurements. Remote Sensing of Environment, 89, 1–28.

[fes3498-bib-0068] Li, Q. , Liu, R. , Wu, T. , & Zhang, M. (2017). Interactions between soluble dietary fibers and wheat gluten in dough studied by confocal laser scanning microscopy. Food Research International, 95, 19–27. 28395821 10.1016/j.foodres.2017.02.021

[fes3498-bib-0069] Lobley, G. E. , Holtrop, G. , Bremner, D. M. , Calder, A. G. , Milne, E. , & Johnstone, A. M. (2013). Impact of short term consumption of diets high in either non‐starch polysaccharides or resistant starch in comparison with moderate weight loss on indices of insulin sensitivity in subjects with metabolic syndrome. Nutrients, 5, 2144–2172. 23752495 10.3390/nu5062144 PMC3725498

[fes3498-bib-0070] Ma, J. , Sun, D.‐W. , Pu, H. , Cheng, J.‐H. , & Wei, Q. (2019). Advanced techniques for hyperspectral imaging in the food industry: Principles and recent applications. Annual Review of Food Science and Technology, 10, 197–220. 10.1146/annurev-food-032818-121155 30633569

[fes3498-bib-0071] Ma, J. , Zheng, B. , & He, Y. (2022). Applications of a hyperspectral imaging system used to estimate wheat grain protein: A review. Frontiers in Plant Science, 13, 837200. 35463397 10.3389/fpls.2022.837200 PMC9024351

[fes3498-bib-0072] Maccaferri, M. , Harris, N. S. , Twardziok, S. O. , Pasam, R. K. , Gundlach, H. , Spannagl, M. , Ormanbekova, D. , Lux, T. , Prade, V. M. , & Milner, S. G. (2019). Durum wheat genome highlights past domestication signatures and future improvement targets. Nature Genetics, 51, 885–895. 30962619 10.1038/s41588-019-0381-3

[fes3498-bib-0073] Mahesh, S. , Manickavasagan, A. , Jayas, D. , Paliwal, J. , & White, N. (2008). Feasibility of near‐infrared hyperspectral imaging to differentiate Canadian wheat classes. Biosystems Engineering, 101, 50–57.

[fes3498-bib-0074] Manley, M. , Du Toit, G. , & Geladi, P. (2011). Tracking diffusion of conditioning water in single wheat kernels of different hardnesses by near infrared hyperspectral imaging. Analytica Chimica Acta, 686, 64–75. 21237309 10.1016/j.aca.2010.11.042

[fes3498-bib-0075] Mariotti, F. , Tomé, D. , & Mirand, P. P. (2008). Converting nitrogen into protein—Beyond 6.25 and Jones' factors. Critical Reviews in Food Science and Nutrition, 48, 177–184. 18274971 10.1080/10408390701279749

[fes3498-bib-0076] Matsuoka, Y. , & Nasuda, S. (2004). Durum wheat as a candidate for the unknown female progenitor of bread wheat: An empirical study with a highly fertile F 1 hybrid with *Aegilops tauschii* Coss. Theoretical and Applied Genetics, 109, 1710–1717. 15448900 10.1007/s00122-004-1806-6

[fes3498-bib-0077] Mazzucotelli, E. , Sciara, G. , Mastrangelo, A. M. , Desiderio, F. , Xu, S. S. , Faris, J. , Hayden, M. J. , Tricker, P. J. , Ozkan, H. , Echenique, V. , Steffenson, B. J. , Knox, R. , Niane, A. A. , Udupa, S. M. , Longin, F. C. H. , Marone, D. , Petruzzino, G. , Corneti, S. , Ormanbekova, D. , … Bassi, F. M. (2020). The global durum wheat panel (GDP): An international platform to identify and exchange beneficial alleles. Frontiers in Plant Science, 11, 569905. 10.3389/fpls.2020.569905 33408724 PMC7779600

[fes3498-bib-0078] McFadden, E. S. , & Sears, E. R. (1946). The origin of Triticum spelta and its free‐threshing hexaploid relatives. Journal of Heredity, 37, 81–89. 20985728 10.1093/oxfordjournals.jhered.a105590

[fes3498-bib-0079] Miller, T. , Ambrose, M. , Reader, S. , Caligari, P. , & Brandham, P. (2001). The Watkins collection of landrace derived wheats. Wheat taxon legacy of John Percival (pp. 113–120). Linnean Society.

[fes3498-bib-0080] Minaei, S. , Shafiee, S. , Polder, G. , Moghadam‐Charkari, N. , van Ruth, S. , Barzegar, M. , Zahiri, J. , Alewijn, M. , & Kuś, P. M. (2017). VIS/NIR imaging application for honey floral origin determination. Infrared Physics & Technology, 86, 218–225.

[fes3498-bib-0081] Moore, K. L. , Schröder, M. , Lombi, E. , Zhao, F.‐J. , McGrath, S. P. , Hawkesford, M. J. , Shewry, P. R. , & Grovenor, C. R. M. (2010). NanoSIMS analysis of arsenic and selenium in cereal grain. New Phytologist, 185, 434–445. 10.1111/j.1469-8137.2009.03071.x 19895416

[fes3498-bib-0082] Müller, J. (2017). Dumas or Kjeldahl for reference analysis. FOSS.

[fes3498-bib-0083] Newberry, M. , Zwart, A. B. , Whan, A. , Mieog, J. C. , Sun, M. , Leyne, E. , Pritchard, J. , Daneri‐Castro, S. N. , Ibrahim, K. , Diepeveen, D. , Howitt, C. A. , & Ral, J. P. F. (2018). Does late maturity alpha‐amylase impact wheat baking quality? Frontiers in Plant Science, 9, 1356. 10.3389/fpls.2018.01356 30245701 PMC6137811

[fes3498-bib-0084] Ngo, P. D. (1999). Energy dispersive spectroscopy. In L. C. Wagner (Ed.), Failure analysis of integrated circuits (pp. 205–215). Springer.

[fes3498-bib-0085] O'Sullivan, A. , O'Connor, B. , Kelly, A. , & McGRATH, M. J. (1999). The use of chemical and infrared methods for analysis of milk and dairy products. International Journal of Dairy Technology, 52, 139–148.

[fes3498-bib-0086] Paltridge, N. G. , Milham, P. J. , Ortiz‐Monasterio, J. I. , Velu, G. , Yasmin, Z. , Palmer, L. J. , Guild, G. E. , & Stangoulis, J. C. (2012). Energy‐dispersive X‐ray fluorescence spectrometry as a tool for zinc, iron and selenium analysis in whole grain wheat. Plant and Soil, 361, 261–269.

[fes3498-bib-0087] Pan, T. T. , Sun, D. W. , Cheng, J. H. , & Pu, H. (2016). Regression algorithms in hyperspectral data analysis for meat quality detection and evaluation. Comprehensive Reviews in Food Science and Food Safety, 15, 529–541. 33401821 10.1111/1541-4337.12191

[fes3498-bib-0088] Payne, P. I. , & Lawrence, G. J. (1983). Catalogue of alleles for the complex gene loci, Glu‐A1, Glu‐B1, and Glu‐D1 which code for high‐molecular‐weight subunits of glutenin in hexaploid wheat (Vol. 11, pp. 29–35). Cereal Research Communications.

[fes3498-bib-0089] Peruchi, L. C. , Nunes, L. C. , de Carvalho, G. G. A. , Guerra, M. B. B. , de Almeida, E. , Rufini, I. A. , Santos, D., Jr. , & Krug, F. J. (2014). Determination of inorganic nutrients in wheat flour by laser‐induced breakdown spectroscopy and energy dispersive X‐ray fluorescence spectrometry. Spectrochimica Acta Part B: Atomic Spectroscopy, 100, 129–136.

[fes3498-bib-0090] Pfitzner, K. , Bartolo, R. , Carr, G. , Esparon, A. , & Bollhöfer, A. (2011). Standards for reflectance spectral measurement of temporal vegetation plots (pp. 1325–1554). Department of Sustainability, Environment, Water, Population and Communities. https://www.dcceew.gov.au/science‐research/supervising‐scientist/publications/ssr/standards‐for‐reflectance‐spectral‐measurement‐of‐temporal‐vegetation‐plots

[fes3498-bib-0091] Pont, C. , Leroy, T. , Seidel, M. , Tondelli, A. , Duchemin, W. , Armisen, D. , Lang, D. , Bustos‐Korts, D. , Goué, N. , Balfourier, F. , et al. (2019). Tracing the ancestry of modern bread wheats. Nature Genetics, 51, 905–911. 10.1038/s41588-019-0393-z 31043760

[fes3498-bib-0092] Poudel, R. , Bhinderwala, F. , Morton, M. , Powers, R. , & Rose, D. J. (2021). Metabolic profiling of historical and modern wheat cultivars using proton nuclear magnetic resonance spectroscopy. Scientific Reports, 11, 1–10. 33542370 10.1038/s41598-021-82616-3 PMC7862595

[fes3498-bib-0093] Pshenichnikova, T. A. , Ermakova, M. F. , Chistyakova, A. K. , Shchukina, L. V. , Berezovskaya, E. V. , Lochwasser, U. , Röder, M. , & Börner, A. (2008). Mapping of the quantitative trait loci (QTL) associated with grain quality characteristics of the bread wheat grown under different environmental conditions. Russian Journal of Genetics, 44, 74–84. 10.1134/S1022795408010109 18409391

[fes3498-bib-0094] Pyler, E. , & Gorton, L. (2008). Baking science & technology: Volume I: Fundamentals & ingredients. Sosland Publishing.

[fes3498-bib-0095] Ravikanth, L. , Jayas, D. S. , White, N. D. , Fields, P. G. , & Sun, D.‐W. (2017). Extraction of spectral information from hyperspectral data and application of hyperspectral imaging for food and agricultural products. Food and Bioprocess Technology, 10, 1–33.

[fes3498-bib-0096] Reale, Á. , Rosati, A. , Tedeschini, E. , Ferri, V. , Cerri, M. , Ghitarrini, S. , Timorato, V. , Ayano, B. , Porfiri, O. , & Frenguelli, G. (2017). Ovary size in wheat (*Triticum aestivum* L.) is related to cell number. Crop Science, 57, 914–925.

[fes3498-bib-0097] Rees, R. , Martin, D. , & Law, D. (1984). Black point in bread wheat: Effects on quality and germination, and fungal associations. Australian Journal of Experimental Agriculture, 24, 601–605.

[fes3498-bib-0098] Reynolds, M. P. , Slafer, G. A. , Foulkes, J. M. , Griffiths, S. , Murchie, E. H. , Carmo‐Silva, E. , Asseng, S. , Chapman, S. C. , Sawkins, M. , Gwyn, J. , & Flavell, R. B. (2022). A wiring diagram to integrate physiological traits of wheat yield potential. Nature Food, 3, 318–324. 10.1038/s43016-022-00512-z 37117579

[fes3498-bib-0099] Robles‐Zazueta, C. A. , Molero, G. , Pinto, F. , Foulkes, M. J. , Reynolds, M. P. , & Murchie, E. H. (2021). Field‐based remote sensing models predict radiation use efficiency in wheat. Journal of Experimental Botany, 72, 3756–3773. 33713415 10.1093/jxb/erab115 PMC8096595

[fes3498-bib-0100] Roy, J. K. , Borah, A. , Mahanta, C. L. , & Mukherjee, A. K. (2013). Cloning and overexpression of raw starch digesting α‐amylase gene from Bacillus subtilis strain AS01a in Escherichia coli and application of the purified recombinant α‐amylase (AmyBS‐I) in raw starch digestion and baking industry. Journal of Molecular Catalysis B: Enzymatic, 97, 118–129.

[fes3498-bib-0101] Ruan, Y. , Yu, B. , Knox, R. E. , Singh, A. K. , DePauw, R. , Cuthbert, R. , Zhang, W. , Piche, I. , Gao, P. , Sharpe, A. , & Fobert, P. (2020). High density mapping of quantitative trait loci conferring gluten strength in Canadian durum wheat. Frontiers in Plant Science, 11, 170. 10.3389/fpls.2020.00170 32194591 PMC7064722

[fes3498-bib-0102] Sáez‐Plaza, P. , Michałowski, T. , Navas, M. J. , Asuero, A. G. , & Wybraniec, S. (2013). An overview of the Kjeldahl method of nitrogen determination. Part I. early history, chemistry of the procedure, and titrimetric finish. Critical Reviews in Analytical Chemistry, 43, 178–223.

[fes3498-bib-0103] Sarić, R. , Nguyen, V. D. , Burge, T. , Berkowitz, O. , Trtílek, M. , Whelan, J. , Lewsey, M. G. , & Čustović, E. (2022). Applications of hyperspectral imaging in plant phenotyping. Trends in Plant Science, 27, 301–315. 10.1016/j.tplants.2021.12.003 34998690

[fes3498-bib-0104] Schuler, S. F. , Bacon, R. K. , Finney, P. L. , & Gbur, E. E. (1995). Relationship of test weight and kernel properties to milling and baking quality in soft red winter wheat. Crop Science, 35, 949–953.

[fes3498-bib-0105] Shahin, M. A. , & Symons, S. J. (2008). Detection of hard vitreous and starchy kernels in amber durum wheat samples using hyperspectral imaging (GRL number M306). NIR News, 19, 16–18.

[fes3498-bib-0106] Sheraz, S. , Wan, Y. , Venter, E. , Verma, S. K. , Xiong, Q. , Waites, J. , Connorton, J. M. , Shewry, P. R. , Moore, K. L. , & Balk, J. (2021). Subcellular dynamics studies of iron reveal how tissue‐specific distribution patterns are established in developing wheat grains. New Phytologist, 231, 1644–1657. 10.1111/nph.17440 33914919

[fes3498-bib-0108] Shewry, P. (2019). What is gluten—Why is it special? Frontiers in Nutrition, 6, 101. 31334243 10.3389/fnut.2019.00101 PMC6625226

[fes3498-bib-0109] Shewry, P. R. (2007). Improving the protein content and composition of cereal grain. Journal of Cereal Science, 46, 239–250.

[fes3498-bib-0110] Shewry, P. R. , Corol, D. I. , Jones, H. D. , Beale, M. H. , & Ward, J. L. (2017). Defining genetic and chemical diversity in wheat grain by 1H‐NMR spectroscopy of polar metabolites. Molecular Nutrition & Food Research, 61, 1600807. 28087883 10.1002/mnfr.201600807 PMC5516129

[fes3498-bib-0111] Shewry, P. R. , D'Ovidio, R. , Lafiandra, D. , Jenkins, J. A. , Mills, E. C. , & Békés, F. (2009). Wheat grain proteins. In Y. Pomeranz (Ed.), Wheat: Chemistry and Technology (pp. 223–298). AACCI.

[fes3498-bib-0112] Shewry, P. R. , Halford, N. G. , Belton, P. S. , & Tatham, A. S. (2002). The structure and properties of gluten: An elastic protein from wheat grain. Philosophical Transactions of the Royal Society of London. Series B: Biological Sciences, 357, 133–142. 11911770 10.1098/rstb.2001.1024 PMC1692935

[fes3498-bib-0113] Shewry, P. R. , & Hey, S. J. (2015). The contribution of wheat to human diet and health. Food and Energy Security, 4, 178–202. 27610232 10.1002/fes3.64 PMC4998136

[fes3498-bib-0114] Shewry, P. R. , Underwood, C. , Wan, Y. , Lovegrove, A. , Bhandari, D. , Toole, G. , Mills, E. C. , Denyer, K. , & Mitchell, R. A. (2009). Storage product synthesis and accumulation in developing grains of wheat. Journal of Cereal Science, 50, 106–112.

[fes3498-bib-0115] Shi, H. , Lei, Y. , Prates, L. L. , & Yu, P. (2019). Evaluation of near‐infrared (NIR) and Fourier transform mid‐infrared (ATR‐FT/MIR) spectroscopy techniques combined with chemometrics for the determination of crude protein and intestinal protein digestibility of wheat. Food Chemistry, 272, 507–513. 30309575 10.1016/j.foodchem.2018.08.075

[fes3498-bib-0116] Simonne, A. , Simonne, E. , Eitenmiller, R. , Mills, H. , & Cresman Iii, C. (1997). Could the dumas method replace the Kjeldahl digestion for nitrogen and crude protein determinations in foods? Journal of the Science of Food and Agriculture, 73, 39–45.

[fes3498-bib-0117] Singh, C. , Jayas, D. , Paliwal, J. , & White, N. (2007). Fungal detection in wheat using near‐infrared hyperspectral imaging. Transactions of the ASABE, 50, 2171–2176.

[fes3498-bib-0118] Singh, C. , Jayas, D. , Paliwal, J. , & White, N. (2009). Detection of insect‐damaged wheat kernels using near‐infrared hyperspectral imaging. Journal of Stored Products Research, 45, 151–158.

[fes3498-bib-0119] Slavin, J. (2003). Why whole grains are protective: Biological mechanisms. Proceedings of the Nutrition Society, 62, 129–134. 12740067 10.1079/PNS2002221

[fes3498-bib-0120] Smale, M. (1997). The green revolution and wheat genetic diversity: Some unfounded assumptions. World Development, 25, 1257–1269.

[fes3498-bib-0121] Stone, B. , & Morell, M. (2009). Carbohydrates: Wheat, chemistry and technology. American Association of Cereal Chemists.

[fes3498-bib-0122] Stone, P. , & Savin, R. (1999). Grain quality and its physiological determinants. In E. H. Satorre & G. A. Slafer (Eds.), Wheat: Ecology and physiology of yield determination (pp. 85–120). Food Product Press.

[fes3498-bib-0123] Su, W.‐H. , He, H.‐J. , & Sun, D.‐W. (2017). Non‐destructive and rapid evaluation of staple foods quality by using spectroscopic techniques: A review. Critical Reviews in Food Science and Nutrition, 57, 1039–1051. 10.1080/10408398.2015.1082966 26480047

[fes3498-bib-0124] Sun, D. , Cen, H. , Weng, H. , Wan, L. , Abdalla, A. , El‐Manawy, A. I. , Zhu, Y. , Zhao, N. , Fu, H. , & Tang, J. (2019). Using hyperspectral analysis as a potential high throughput phenotyping tool in GWAS for protein content of rice quality. Plant Methods, 15, 1–16. 31139243 10.1186/s13007-019-0432-x PMC6532189

[fes3498-bib-0125] Tahmasbian, I. , Morgan, N. K. , Hosseini Bai, S. , Dunlop, M. W. , & Moss, A. F. (2021). Comparison of hyperspectral imaging and near‐infrared spectroscopy to determine nitrogen and carbon concentrations in wheat. Remote Sensing, 13, 1128.

[fes3498-bib-0126] Takeda, Y. , Hizukuri, S. , & Juliano, B. O. (1986). Purification and structure of amylose from rice starch. Carbohydrate Research, 148, 299–308.

[fes3498-bib-0127] Tan, C. W. , Wang, D. L. , Zhou, J. , Du, Y. , Luo, M. , Zhang, Y. J. , & Guo, W. S. (2018). Assessment of F(v)/F(m) absorbed by wheat canopies employing in‐situ hyperspectral vegetation indexes. Scientific Reports, 8, 9525. 10.1038/s41598-018-27902-3 29934625 PMC6015031

[fes3498-bib-0128] Taneva, K. , Bozhanova, V. , & Petrova, I. (2019). Variability, heritability and genetic advance of some grain quality traits and grain yield in durum wheat genotypes. Bulgarian Journal of Agricultural Science, 25, 288–295.

[fes3498-bib-0129] Thorwarth, P. , Piepho, H. P. , Zhao, Y. , Ebmeyer, E. , Schacht, J. , Schachschneider, R. , Kazman, E. , Reif, J. C. , Würschum, T. , & Longin, C. F. H. (2018). Higher grain yield and higher grain protein deviation underline the potential of hybrid wheat for a sustainable agriculture. Plant Breeding, 137, 326–337. 10.1111/pbr.12588

[fes3498-bib-0130] Tsirivakou, A. , Melliou, E. , & Magiatis, P. (2020). A method for the rapid measurement of Alkylresorcinols in flour, bread and related products based on 1H qNMR. Food, 9, 1025. 10.3390/foods9081025 PMC7466349 32751799

[fes3498-bib-0131] Turnbull, K.‐M. , & Rahman, S. (2002). Endosperm texture in wheat. Journal of Cereal Science, 36, 327–337.

[fes3498-bib-0132] Uauy, C. , Brevis, J. C. , & Dubcovsky, J. (2006). The high grain protein content gene Gpc‐B1 accelerates senescence and has pleiotropic effects on protein content in wheat. Journal of Experimental Botany, 57, 2785–2794. 16831844 10.1093/jxb/erl047

[fes3498-bib-0133] Uauy, C. , Distelfeld, A. , Fahima, T. , Blechl, A. , & Dubcovsky, J. (2006). A NAC gene regulating senescence improves grain protein, zinc, and iron content in wheat. Science, 314, 1298–1301. 17124321 10.1126/science.1133649 PMC4737439

[fes3498-bib-0134] Veraverbeke, W. S. , & Delcour, J. A. (2002). Wheat protein composition and properties of wheat glutenin in relation to breadmaking functionality. Critical Reviews in Food Science and Nutrition, 42, 179–208. 12058979 10.1080/10408690290825510

[fes3498-bib-0135] Vigneau, N. , Ecarnot, M. , Rabatel, G. , & Roumet, P. (2011). Potential of field hyperspectral imaging as a non destructive method to assess leaf nitrogen content in wheat. Field Crops Research, 122, 25–31.

[fes3498-bib-0136] Wang, K. , & Fu, B. X. (2020). Inter‐relationships between test weight, thousand kernel weight, kernel size distribution and their effects on durum wheat milling, semolina composition and pasta processing quality. Food, 9, 1308. 10.3390/foods9091308 PMC7555757 32948041

[fes3498-bib-0137] Ward, M. H. (2009). Too much of a good thing? Nitrate from nitrogen fertilizers and cancer: President's cancer panel‐October 21, 2008. Reviews on Environmental Health, 24, 357–363. 20384045 10.1515/reveh.2009.24.4.357 PMC3068045

[fes3498-bib-0138] Warechowska, M. , Markowska, A. , Warechowski, J. , Miś, A. , & Nawrocka, A. (2016). Effect of tempering moisture of wheat on grinding energy, middlings and flour size distribution, and gluten and dough mixing properties. Journal of Cereal Science, 69, 306–312.

[fes3498-bib-0139] Watson, M. , & Galliher, T. (2001). Comparison of dumas and Kjeldahl methods with automatic analyzers on agricultural samples under routine rapid analysis conditions. Communications in Soil Science and Plant Analysis, 32, 2007–2019.

[fes3498-bib-0140] Wieser, H. (2007). Chemistry of gluten proteins. Food Microbiology, 24, 115–119. 17008153 10.1016/j.fm.2006.07.004

[fes3498-bib-0141] Wieser, H. , Antes, S. , & Seilmeier, W. (1998). Quantitative determination of gluten protein types in wheat flour by reversed‐phase high‐performance liquid chromatography. Cereal Chemistry, 75, 644–650.

[fes3498-bib-0142] Wieser, H. , Bushuk, W. , & MacRitchie, F. (2006). The polymeric glutenins. In Gliadin and Glutenin: The Unique Balance of Wheat Quality (pp. 213–240). American Association of Cereal Chemists, Inc (AACC).

[fes3498-bib-0143] Wieser, H. , Koehler, P. , & Scherf, K. A. (2022). Chemistry of wheat gluten proteins: Qualitative composition. Cereal Chemistry, 100, 23–35. 10.1002/cche.10572

[fes3498-bib-0144] Wilschefski, S. C. , & Baxter, M. R. (2019). Inductively coupled plasma mass spectrometry: Introduction to analytical aspects. The Clinical Biochemist. Reviews, 40, 115–133. 10.33176/aacb-19-00024 31530963 PMC6719745

[fes3498-bib-0145] Wu, B. , Andersch, F. , Weschke, W. , Weber, H. , & Becker, J. S. (2013). Diverse accumulation and distribution of nutrient elements in developing wheat grain studied by laser ablation inductively coupled plasma mass spectrometry imaging. Metallomics: Integrated Biometal Science, 5, 1276–1284. 10.1039/c3mt00071k 23877092

[fes3498-bib-0146] Yoon, S.‐C. , Lawrence, K. , Siragusa, G. , Line, J. , Park, B. , & Feldner, P. (2009). Hyperspectral reflectance imaging for detecting a foodborne pathogen: Campylobacter. Transactions of the ASABE, 52, 651–662.

[fes3498-bib-0147] Yoon, S.‐C. , & Park, B. (2015). Hyperspectral image processing methods. In Hyperspectral imaging Technology in Food and Agriculture (pp. 81–101). Springer.

[fes3498-bib-0148] Zhao, H. , Song, X. , Yang, G. , Li, Z. , Zhang, D. , & Feng, H. (2019). Monitoring of nitrogen and grain protein content in winter wheat based on sentinel‐2A data. Remote Sensing, 11, 1724.

[fes3498-bib-0149] Zhou, H. , Riche, A. B. , Hawkesford, M. J. , Whalley, W. R. , Atkinson, B. S. , Sturrock, C. J. , & Mooney, S. J. (2021). Determination of wheat spike and spikelet architecture and grain traits using X‐ray computed tomography imaging. Plant Methods, 17, 26. 10.1186/s13007-021-00726-5 33750418 PMC7945051

[fes3498-bib-0150] Zhou, X. , Kono, Y. , Win, A. , Matsui, T. , & Tanaka, T. S. (2021). Predicting within‐field variability in grain yield and protein content of winter wheat using UAV‐based multispectral imagery and machine learning approaches. Plant Production Science, 24, 137–151.

